# Aldose reductase inhibitor form *Cassia glauca*: A comparative study of cytotoxic activity with Ag nanoparticles (NPs) and molecular docking evaluation

**DOI:** 10.1371/journal.pone.0240856

**Published:** 2020-10-16

**Authors:** Samir M. Osman, Nahla A. Ayoub, Safaa A. Hafez, Haitham A. Ibrahim, Mohamed A. El Raey, Soad Z. El-Emam, Ahmed A. Seada, Amr M. Saadeldeen

**Affiliations:** 1 Department of Pharmacognosy, Faculty of Pharmacy, October 6 University, Giza, Egypt; 2 Department of Pharmacognosy, Faculty of Pharmacy, Ain Shams University, Cairo, Egypt; 3 Department of Pharmacology and Toxicology, Faculty of Medicine, Umm Al Qura University, Makkah, Saudi Arabia; 4 Department of Pharmacognosy, Faculty of Pharmacy, Helwan University, Cairo, Egypt; 5 Department of Phytochemistry and Plant Systematics, National Research Center, Dokki, Giza, Egypt; 6 Department of Pharmacology and Toxicology, Faculty of Pharmacy, October 6 University, Giza, Egypt; 7 Department of Pharmacognosy, Faculty of Pharmacy, Cairo University, Cairo, Egypt; 8 Department of Pharmacognosy, School of Pharmacy, Newgiza University, Giza, Egypt; Institute of medical research and medicinal plant studies, CAMEROON

## Abstract

UPLC-MS/MS profiling of *Cassia glauca* leaves extract revealed the identification of 10 flavonoids. Kaempferol 3-*O*-*β*-D-rutinoside was isolated and studied for its cytotoxic activity. It showed high cytotoxic effects against MCF-7 (IC_50_ of 4.6±0.038 μg/ml) and HepG-2 (IC_50_ of 8.2±0.024 μg/ml) cancer cell lines, compared to the leaves extracts, their Ag nanoparticles, and doxorubicin. Moreover, Kaempferol 3-*O*-*β*-D-rutinoside exerted a synergistic cytotoxic effect with doxorubicin on MCF-7 cell lines. It was discovered as kinases and aldose reductase inhibitor while rationalizing its cytotoxic activity through molecular docking study. Thus, it is expected that the cardiotoxic effects of doxorubicin can be also decreased by using Kaempferol 3-*O*-*β*-D-rutinoside due to its aldose reductase inhibitory effect. These findings suggested that Kaempferol 3-*O*-*β*-D-rutinoside could be used in combination with chemotherapeutic drugs to increase the sensitivity to their cytotoxic activity and protect against their side effects.

## Introduction

The green synthesis of nanoparticles was recently much considered. Silver has been used for various therapeutic purposes. The AgNPs provides a simple, cheap, and safe therapeutic products with less adverse effects. Cytotoxic effects of AgNPs were proved on various cancer cells. Currently, several plant extracts have been used for green synthesis purposes [1, 2].

Cassia, a major tropical genus belonging to the Caesalpiniaceae, comprising about 600 species. *Cassia glauca* Lam. (syn. *Cassia surattensis* Burm. f.) is native to India, tropical Asia, and Australia. The species characterized by being glabrous, evergreen, and fast-growing shrubs to small trees, each about 2-5m height. Leaves are bipinnately compound with 4–6 pairs of ovate leaflets with emarginate apices. Flowers are brightly yellow colored arranged in axillary racemes [3].

*Cassia glauca* has been widely used traditionally for treating diabetes, gonorrhea, and blennorrhagia. Furthermore, it was traditionally used for its central depressant, diuretic, antimalarial, and purgative effects [3–5]. Different extracts of *Cassia glauca* Lam. were previously evaluated for several pharmacological activities. Alcoholic and water extracts of the aerial parts and seeds of *Cassia glauca* were evaluated for their antioxidant activity [6–10]. They were also reported to have antidiabetic effects due to α-amylase and α-glucosidase enzymes inhibitory effects [6, 11–13]. Leaves and seeds extracts showed hepatoprotective activity against CCl_4_-induced and paracetamol-induced liver toxicity in rats [8, 10] and also possessed broad-spectrum antimicrobial activity [7, 14]. The cytotoxic activity of the plant was previously screened against several cancer cell lines [10, 15, 16]. The plant was found to be rich in anthraquinones; aloe-emodin, physcion, and chrysophanol, as well as flavonoids including apigenin, luteolin, quercetin, kaempferol, and their glycosides [4, 10, 13, 15]. The high phenolic content characterizing *Cassia glauca* increased our expectation about using the plant extract as a natural reducing and capping agent for green synthesis of AgNPs [1].

The role of inhibition of aldose reductase in cancer management was investigated and proven through inhibition of aldose reductase-mediated ROS signaling and preventing activation of various oncogenic kinases and transcription factors that are responsible for the production of several carcinogenic mediators [17, 18]. The use of doxorubicin, a potent chemotherapeutic drug against cancers, became limited due to its cardiotoxic effects [19–21]. Aldose reductase inhibitors showed a potential increase in the cytotoxic activity of doxorubicin with a reduction in its cardiotoxicity [22, 23].

Thus, the current study was designed for isolation of possible pure natural aldose reductase inhibitors from *Cassia glauca*, confirmed via molecular docking study, and investigation of their cytotoxic activity compared to leaf extracts and their AgNPs. Furthermore, the effect of these compounds on the cytotoxic activity of doxorubicin was also studied.

## Materials and methods

### Herbal material

Leaves of *Cassia glauca* Lam. were collected in May 2016 from the Orman Gardens, Giza, Egypt, and the identity were confirmed by Flora and Phytotaxonomy Research Department, Horticultural Researches Institute, Agricultural Research Centre, Cairo, Egypt.

### UPLC-MS/MS profiling

100 g. of shade-dried powdered *Cassia glauca* leaves were extracted in Soxhlet with 500mL methanol for 30 minutes. The methanol extract was left to cool, filtered and the filtrate was evaporated using Rotavapor^®^ R-100, Buchi, Switzerland. One gram of the methanol extract was sonicated with 200mL 70% acetone, filtered, the residue was further sonicated in 10mL methanol and then centrifuged (1000 rpm, 10 minutes). The supernatant was finally filtered via a membrane filter. Identification was performed using Dionex UltiMate 3000 UPLC System (Thermo Fisher Scientific, Bremen, Germany) equipped with ACQUITY UPLC HSS T3 Column (1.0×150 mm; 1.8 μm) and a photodiode array detector (220–600 nm). Elution was carried out using water and acetonitrile (150 μL/min), effluents were transferred into a hybrid linear ion trap (LIT)–orbital trap (Orbitrap) mass spectrometer (Orbitrap Elite, Thermo Fisher Scientific, Bremen, Germany) equipped with a heated electrospray (HESI) ion source. Peaks assignments were confirmed by authentic samples. The data were evaluated by Xcalibur 2.2 SP1software [24, 25].

### Extraction, fractionation, isolation, and structural elucidation of pure compounds

Two Kg. of shade-dried powdered *Cassia glauca* leaves were macerated with 20 liters of distilled water for 48 hours. The aqueous macerate was filtered, and the filtrate was evaporated to dryness. The aqueous extract (200 g.) was further macerated with methanol (3×2 liters) at 40°C each for 30 minutes. The methanol macerate was left to cool, filtered, and evaporated to dryness. 30 g. of methanol fraction was fractionated on Día-ion Hp-20 (5**×**150 cm) (Sigma Chemical Co., St. Louis, MO, USA) and eluted with different concentrations of water and methanol yielded five collective fractions that were evaporated to dryness under vacuum. Fraction 2; eluted by water 75: methanol 25. was further chromatographed on a Silica gel 60 column (2×150 cm; with particle size 0.063–0.2 and surface area 70–230 mesh ASTM) (Sigma Chemical Co., St. Louis, MO, USA) and eluted with various ratios of ethyl acetate/methanol mixture. The sub-fraction eluted by ethyl acetate 70: methanol 30 was chromatographed on Sephadex LH-20 column (2×50 cm; 25–100 μm) (Sigma Chemical Co., St. Louis, MO, USA) using methanol resulted in the isolation of 13mg and 10mg of compound 1 and 2, respectively. All fractions, sub-fractions, and pure compounds were screened by TLC using ethyl acetate (100): formic acid (11): acetic acid (11): water (20) on pre-coated silica gel 60 on aluminum sheets (E-Merck, Darmstadt, Germany). NMR spectra (^1^H and ^13^C APT) were measured using JEOL JNM-ECA (400 MHz, DMSO-*d*_*6*_) (Faculty of Pharmacy, Ain Shams University, Egypt) while, ESI-MS was performed using Micromass Quattro II (Ruprecht-Karls-Universität, Heidelberg, Germany) [26–28]. The samples were dissolved in DMSO, run at room temperature, and chemical shifts were given as δ ppm relative to tetramethylsilane (TMS) as an internal standard.

### Green synthesis (synthesis of silver nanoparticles; Ag NPs)

40 mg of each of the total aqueous extract, methanol, and remaining aqueous fractions of the leaves of *Cassia glauca* were dissolved in 10mL of the corresponding solvent (stoke solutions). Silver nanoparticles were prepared by the reduction of 10mL aqueous AgNO_3_ solution (1mM) with serial dilutions prepared from *Cassia glauca* extracts stoke solutions (100–500 μl). The mixtures were incubated in the dark for ninety minutes. Ag NPs were purified by centrifugation (10,000 rpm / 20 minutes). Successful formation of Ag NPs was indicated by characteristic color changes which range from yellowish-brown to reddish and deep brown; the UV-1601 PC, UV-visible spectrophotometer (Shimadzu, Kyoto, Japan) was used to determine the band metal wavelengths. The shape and sizes of the prepared Ag NPs were determined using a transmission electron microscope (TEM) (JEOL-JEM-2100, Japan) [29].

### *In vitro* cytotoxic activity

Cytotoxic activity of the total aqueous extract, methanol, and remaining aqueous fractions as well their prepared Ag NPs and the isolated compounds (Kaempferol 3-*O*-*β*-D-rutinoside and Rutin) were tested *in vitro* against human hepatoma (HepG2) and human breast adenocarcinoma (MCF-7) cell lines (supplied by VACSERA, El-Dokki, Giza, Egypt). Cytotoxic activity was investigated *via* cell viability testing by MTT assay as described by Meerloo, *et*.*al*., 2011 [30]. 100 μg/ml doxorubicin and 0.5% DMSO were used as positive and negative controls, respectively [31–33]. The absorbance was determined photometrically using ELISA microplate reader (FLUOstar Omega, BMG, Labtech, Germany) at 570 nm. The relative viability percentage and IC_50_ were calculated according to Mosmann, 1983 [34].

### Evaluation of cell death mode

The effects of Kaempferol 3-*O*-*β*-D-rutinoside, Doxorubicin, and their combination on the cell death mode of human breast adenocarcinoma (MCF-7) cell line were investigated using the method reported by Ciniglai, *et*.*al*., 2010 [35]. Data were represented as a percentage of viable, apoptotic, and necrotic cells population.

### Molecular docking study

ChemBioDraw Ultra 16.0v, PerkinElmer Inc., USA, and MOE^®^ version 2014.09, Chemical Computing Group Inc., Montreal, Canada software were used for sketching compounds and molecular docking studies, respectively. X-ray crystallographic structure of p90 ribosomal S6 kinase (RSK2 kinase; PDB: 3UBD), and aldose reductase (AKR1B1; PDB: 2IKI) was obtained from the RCSB-PDB Protein data bank. All crystallographic water molecules were removed. The crystallographic disorders and the unfilled valence atoms were corrected using protein report and utility, and clean protein options. Then, the complex was submitted to a series of protein-energy minimizations using CHARMM and MMFF94x force fields. The rigid binding site of the protein was obtained by applying fixed atom constraint. The essential amino acids were defined and prepared for the molecular docking process. The molecular docking algorithm was initially validated by redocking of the co-crystallized ligands (SL0101 to RSK2, and IDD388 to AKR1B1). The molecular docking studies were carried out using CDOCKER protocol. ‘Triangle Matcher’ is a method used to define binding sites. It is using three different scoring functions (Affinity dG, London dG, GBVI/WSA), where the poses are produced by overlapping triplets of ligand atoms and triplets of receptor site particles. The receptor site particles are alpha ball centers that demonstrate places of stuffing. Thirty poses were produced for each tested ligand. Duplicate poses were removed: complexes are duplicates if ligand-receptor atom pairs are involved in hydrogen bond and hydrophobic interactions. Then generated poses are scored based on the London dG scoring function, which predicts the binding free energy of the compound from a given pose after steps of refinement, solvation effects were calculated using the GBVI/WSA dG scoring function with the Generalized Born solvation model (GBVI). The GBVI/WSA dG is a forcefield-that estimate the binding energy of the ligand from a given pose [36, 37]. The receptors were held rigid while ligands were allowed to be flexible during the refinement. Each molecule was allowed to produce ten different interaction poses with the active site at the protein. Then, docking scores (-CDOCKER interaction energy) of the best-fitted poses were recorded [38–43].

### Statistical analysis

The statistical analysis was performed using GraphPad Prism software version 6.01 (GraphPad Software Inc., CA, USA). Data were presented as mean ± standard error and/or standard deviation. Statistical significance was determined using one-way ANOVA following with a Tukey-Kramer post hoc test for evaluating the differences between groups. The statistically significant level was established at p-value < 0.05.

## Results and discussions

### UPLC-MS/MS profiling

**Fig 1:**
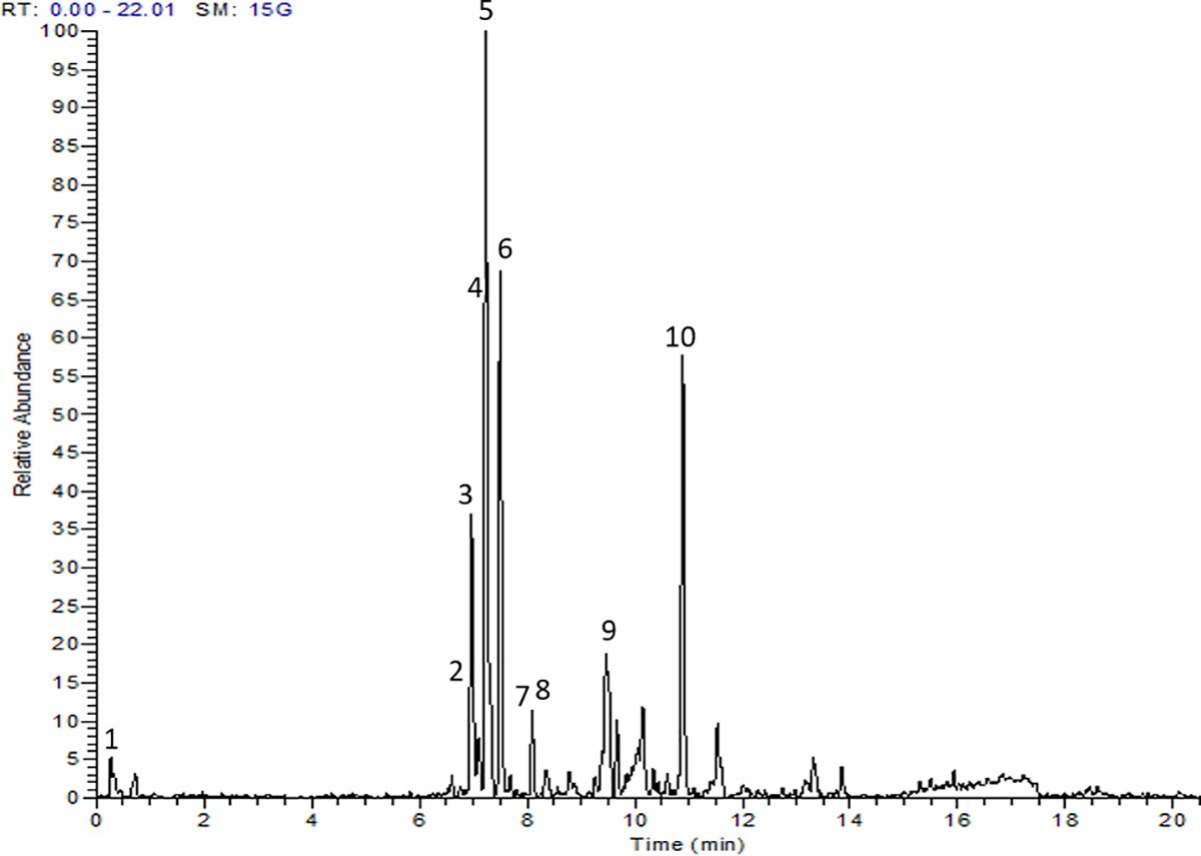
UPLC chromatogram of the methanol extract of *Cassia glauca* leaves.

The methanol extracts obtained from *Cassia glauca* leaves were analyzed by UPLC system for 20 minutes run (Fig 1). Effluents were coupled with a mass spectrometer and 10 compounds were identified based on previous criteria (Table 1). Quercetin glucoside showed the highest abundance (100 mg/15g), followed by kaempferol rutinoside (69 mg/15g), rutin (64.5 mg/15g), and apigenin (58 mg/15g). MS/MS spectra of the major identified compounds are supplemented as S1–S4 Figs.

**Table 1:** Phenolics identified in *Cassia glauca* leaves extract by UPLC-MS/MS.

Peak no.	Rt. (min.)	Relative abundance (mg/15g)	λmax (nm)	[M-H]- (*m/z*)	Major fragments (*m/z*)	MW (g/mol)/	Identified compound
						molecular formula	
**1.**	0.30	5.5	222, 298, 369	341.10870	**179.06**	342.3/	Caffeoyl O-glucoside [44]
					[M-H- glucosyl]^-^	C_15_H_18_O_9_	
					**161.05**		
					[M-H- glucose]		
					^-^ **131.04**		
					[M-H-glucosyl-CO]^-^		
**2.**	6.95	14.5	255, 352	755.20233	**609.15**	756.7/	Quercetin rhamnosyl-rutinoside [45, 46]
					[M-H-rhamnose]^-^	C_33_H_40_O_20_	
					**489.10**		
					[M-H-rhamnose- H_2_O]^-^		
					**300.03**		
					[M-H-2rhamnose- glucose-H]^-^		
**3.**	7.17	37	265, 345	739.20795	**593.15**	740/	Kaempferol rhamnosyl-rutinoside [45, 46]
					[M-H-rhamnose]^-^	C_33_H_40_O_19_	
					**575.14**		
					[M-H-rhamnose- H_2_O]^-^		
					**284.03**		
					[M-H-2rhamnose- glucose-H]^-^		
**4.**	7.22	64.5	255, 355	609.14508	**469.01**	610/	Quercetin rhamnosyl-glucoside (Rutin) [45,46]
					[M-H-rhamnose]^-^	C_27_H_30_O_16_	
					**301.04**		
					[M-H-rhamnose- glucose]^-^		
**5.**	7.31	100	268, 351	463.08765	**301.04**	464.4/	Quercetin glucoside [47]
					[M-H-glucose]^-^	C_21_H_20_O_12_	
**6.**	7.49	69	266, 348	593.15015	**285.04**	594.5/	Kaempferol rutinoside [45, 46]
					[M-H-rutinoside]^-^	C_27_H_30_O_15_	
**7.**	7.57	7.5	268, 348	447.09262	**285**	448.4/	Kaempferol glucoside [48]
					[M-H-glucose]^-^	C_21_H_20_O_11_	
**8.**	8.57	12	206, 274	315.05121	**300.03**	316.3/	Isorhamnetin [49]
					[M-H-CH_3_] –	C_16_H_12_O_7_	
**9.**	9.49	18.5	288, 336	287.22226	**269.21**	288.3/	Eriodictyol [50]
					[M-H-H_2_O] –	C_15_H_12_O_6_	
**10.**	10.87	58	297, 324	269.04523	**241.05**	270.24/	Apigenin [51]
					[M-H-CO]^-^	C_15_H_10_O_5_	
					**225.06**		
					[M-H-CO_2_]^-^		

### Structural elucidation of the isolated pure compounds

Compound (**1**) was obtained as a yellowish-brown amorphous powder (13mg). Its TLC chromatogram showed a dark purple spot under UV-light with an R_f_ value of 0.6 giving a deep green color with FeCl_3_. ESI-MS (negative and positive) spectra that showed [M-H]^-^ at 593.15 *m/z*, and [M+H]^+^ at 595.17 *m/z*, respectively, supplemented as S5 and S6 Figs, together with the NMR (^1^H and ^13^C APT) spectral data, supplemented as S7 and S8 Figs, were indicative for kaempferol 3-O-α-L-rhamnopyranosyl-(1→6)-β-D-glucopyranoside (kaempferol 3-*O*-*β*-D-rutinoside) [52, 53].

Compound (**2**) was isolated as a yellowish-brown amorphous powder (10mg), showed a dark purple spot under UV-light with R_f_ value of 0.36 giving a deep green color with FeCl_3_. ESI-MS (negative and positive) spectra that showed [M-H]^-^ at 609.15 *m/z*, and [M+H]^+^ at 611.16 *m/z*, respectively, supplemented as S9 and S10 Figs, together with ^1^H NMR and ^13^C NMR APT spectral data, supplemented as S11 and S12 Figs, were consistent with those of quercetin-3-O-α-L-rhamnosyl-(1→6)-β-D-glucoside (rutin) [54, 55].

### Synthesis of silver nanoparticles; Ag NPs

**Fig 2:**
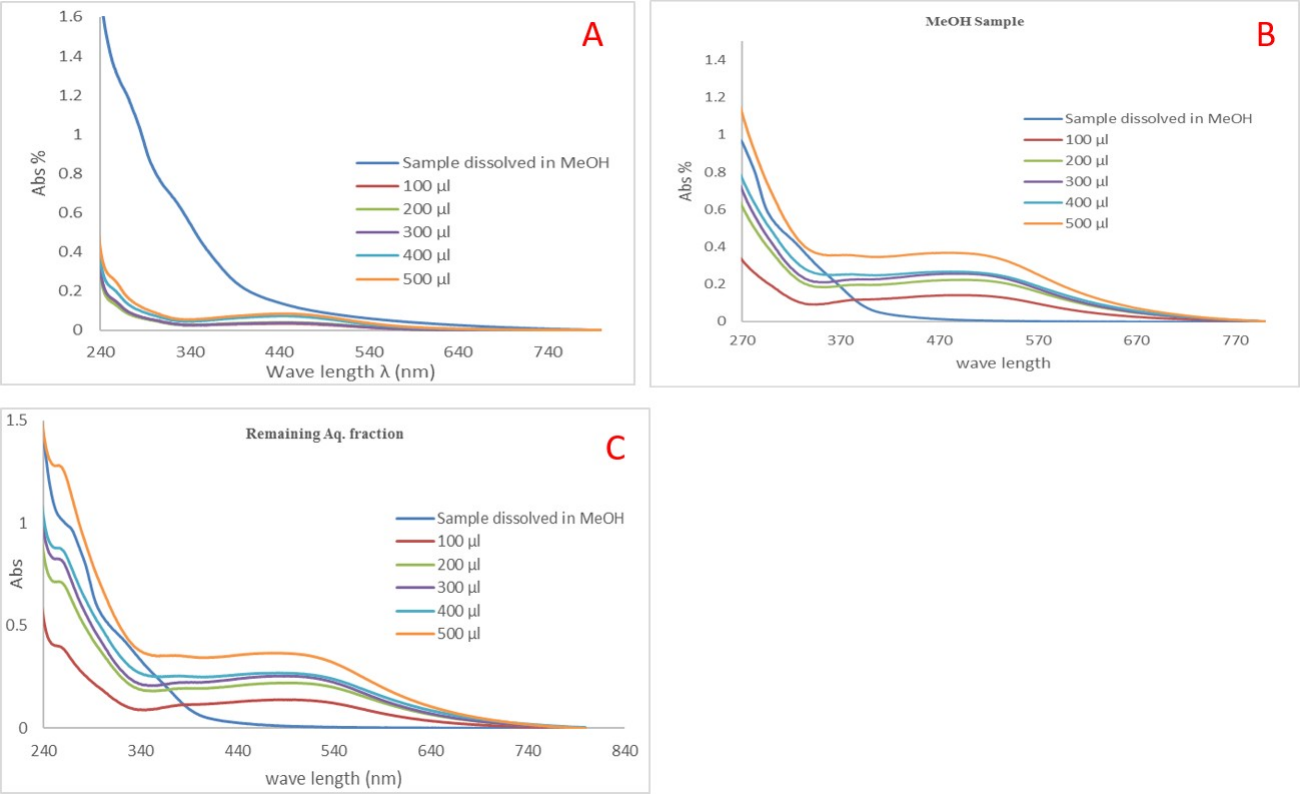
UV-Vis spectra of Ag NPs prepared using (A) total aqueous extract (B) methanol fraction (C) remaining aqueous fraction.

**Fig 3:**
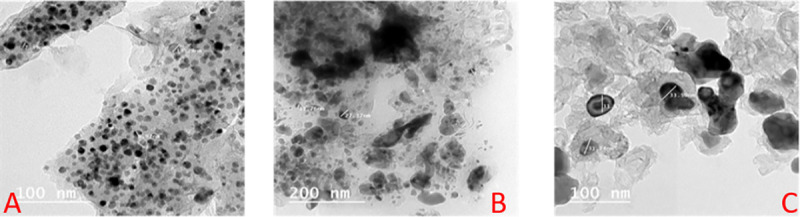
TEM images of Ag NPs prepared using (A) total aqueous extract (B) methanol fraction (C) remaining aqueous fraction.

The yellowish-brown color appeared has been taken as evidence for the formation of Ag NPs due to the surface plasmon resonance (SPR) [56]. The UV-vis spectra of Ag NPs prepared with different concentrations (100, 200, 300, 400, and 500 μL/10 mL of 10^−3^ M AgNO_3_ solution) of the total aqueous extract of *Cassia glauca* leaves, the methanol, and the remaining aqueous fractions showed absorption in the visible region at 457–515 nm due to the SPR band (Fig 2). The increase in the intensity of the SPR band indicates that more Ag^+^ ions are reduced to Ag NPs. Increasing the concentration of the extract means that there are a large number of functional groups available for reduction and capping of the Ag NPs, hence the reaction rate increased and a faster reduction of Ag^+^ ions which in turn enhanced the nucleation rate. Thus, the blue shift of the SPR is a consequence of the formation of smaller Ag NPs. Further increasing in extracts concentrations enhanced the growth rate to produce bigger particles which are reflected in the redshift of the SPR [57]. There was no obvious change in peak position for 10 days, except for the increase of absorbance which indicated the increase in the Ag NPs amount. The stable position of the absorbance peak indicated that new particles did not aggregate. These spectra demonstrated that Ag NPs colloidal solution could be remaining stable within the biological assays [58, 59]. The observed TEM images analysis confirmed the successful formation of Ag NPs with an average diameter range of 10–16 nm (Fig 3). The particles were found separated from each other which reflects the capping action of the plant extracts in the preparation process.

### *In vitro* cytotoxic activity and evaluation of cell death mode

The total aqueous extract, methanol, and remaining aqueous fractions as well their prepared Ag NPs and the isolated compound (Kaempferol 3-*O*-*β*-D-rutinoside and Rutin) were tested *in vitro* for their possible cytotoxic activity against HepG2 and MCF-7 cell lines using MTT assay. Kaempferol 3-*O*-*β*-D-rutinoside; isolated from *Cassia glauca* leaves showed the highest cytotoxic activity among all the tested samples against HepG2 and MCF-7 cell lines with IC_50_ values of 8.2 ± 0.024 and 4.6 ± 0.038 μg/mL, respectively. Rutin showed low potentials for the cytotoxic activity against HepG2 and MCF-7 cell lines with IC_50_ values of 52.22 ± 0.030 and 52.96 ± 0.040 μg/mL, respectively. Furthermore, the synthesized Ag NPs via methanol fraction showed high cytotoxic activity against HepG2 and MCF-7 cell lines with IC_50_ values of 11.21 ± 0.007 and 15.8 ± 0.022 μg/mL, respectively. The other samples of synthesized Ag NPs showed cytotoxic activity higher than the corresponding plant extract samples (Table 2). Compared to doxorubicin, the isolated Kaempferol 3-*O*-*β*-D-rutinoside and the prepared Ag NPs from the leaf extracts of *Cassia glauca* possessed a substantial cytotoxic activity against HepG2 and MCF-7 cell lines.

**Table 2:** Cytotoxic activity (IC_50_ values in μg/mL ± SE) of the total aqueous extract, methanol, remaining aqueous fractions, synthesized Ag NPs and the isolated compound (Kaempferol 3-O-β-D-rutinoside and Rutin) from *Cassia glauca* leaves against HepG2 and MCF-7 cell lines using MTT assay, compared to doxorubicin.

Cell line	IC_50_ (μg/mL)								
	*Cassia glauca* leaves extracts			Synthesized Ag NPs by			Isolated Compounds		Dox.
	Total extract	Methanol fraction	Remaining aqueous fraction	Total extract	Methanol fraction	Remaining aqueous fraction	Kaempferol 3-O-β-D- rutinoside	Rutin	
**HepG2**	28.7	55.4	105.1	16.8	11.21	49.8	8.2	52.22	0.53
	± 0.022	± 0.009	± 0.094	± 0.016	± 0.007	± 0.030	± 0.024	± 0.030	± 0.043
**MCF-7**	88.2	40.8	52.6	22.6	15.8	28.0	4.6	52.96	0.70
	± 0.083	± 0.019	± 0.091	± 0.020	± 0.022	± 0.011	± 0.038	± 0.040	± 0.040

**Fig 4:**
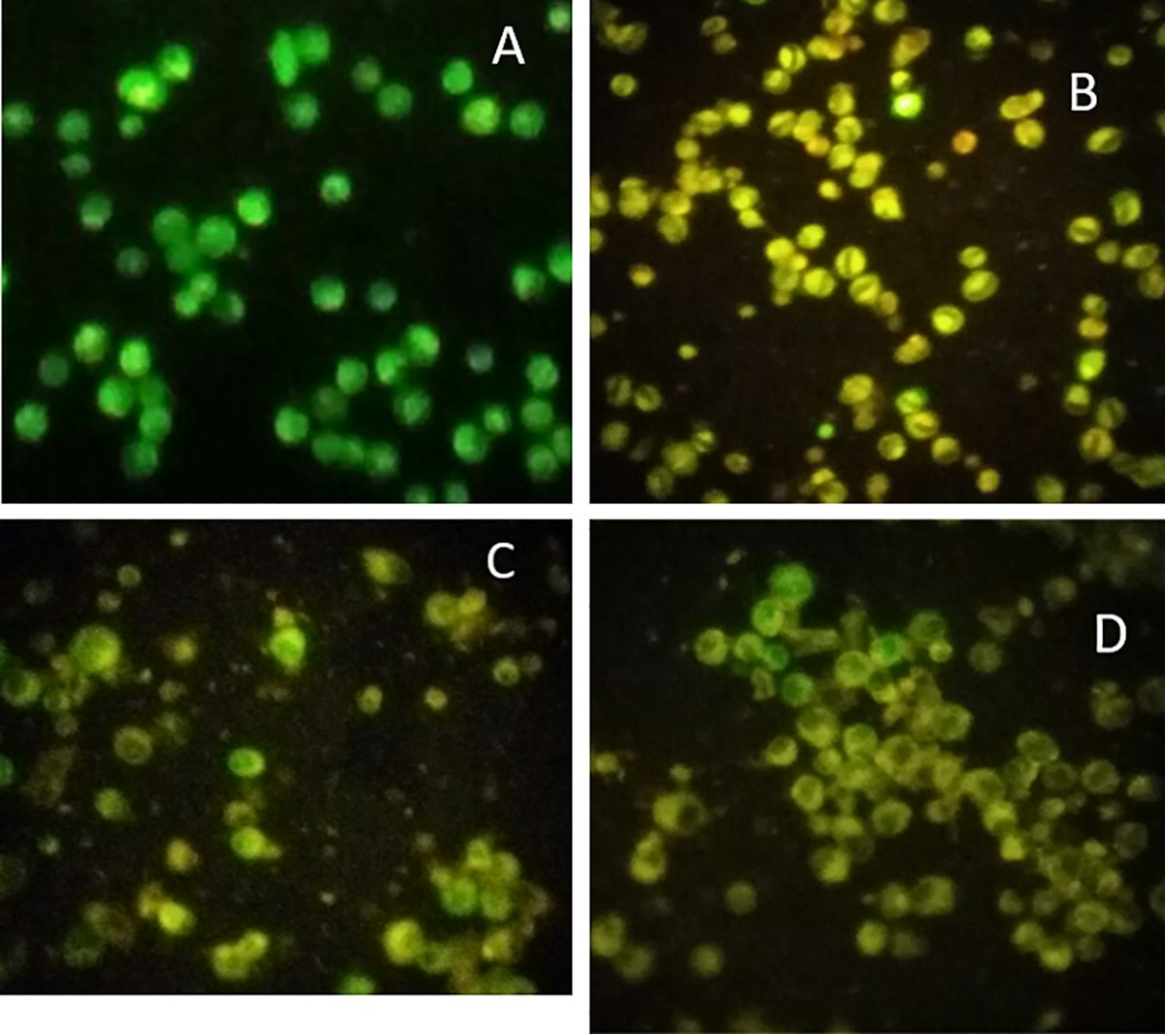
AO/EB staining pattern of different MCF-7 cell populations previously incubated with (A) control (no drugs) (B) doxorubicin (Dox.) (C) kaempferol 3-*O*-*β*-D-rutinoside (D) doxorubicin/kaempferol 3-*O*-*β*-D-rutinoside combination.

The effects of Kaempferol 3-*O*-*β*-D-rutinoside (10 μg/mL), doxorubicin (3 μg/mL), and their combination on the cell death mode of MCF-7 cell line were investigated using fluorescent AO/EB double staining assay. Viable, apoptotic, and necrotic cell populations were detected according to Ciniglai, *et*.*al*., 2010 [35] (Fig 4).

**Fig 5:**
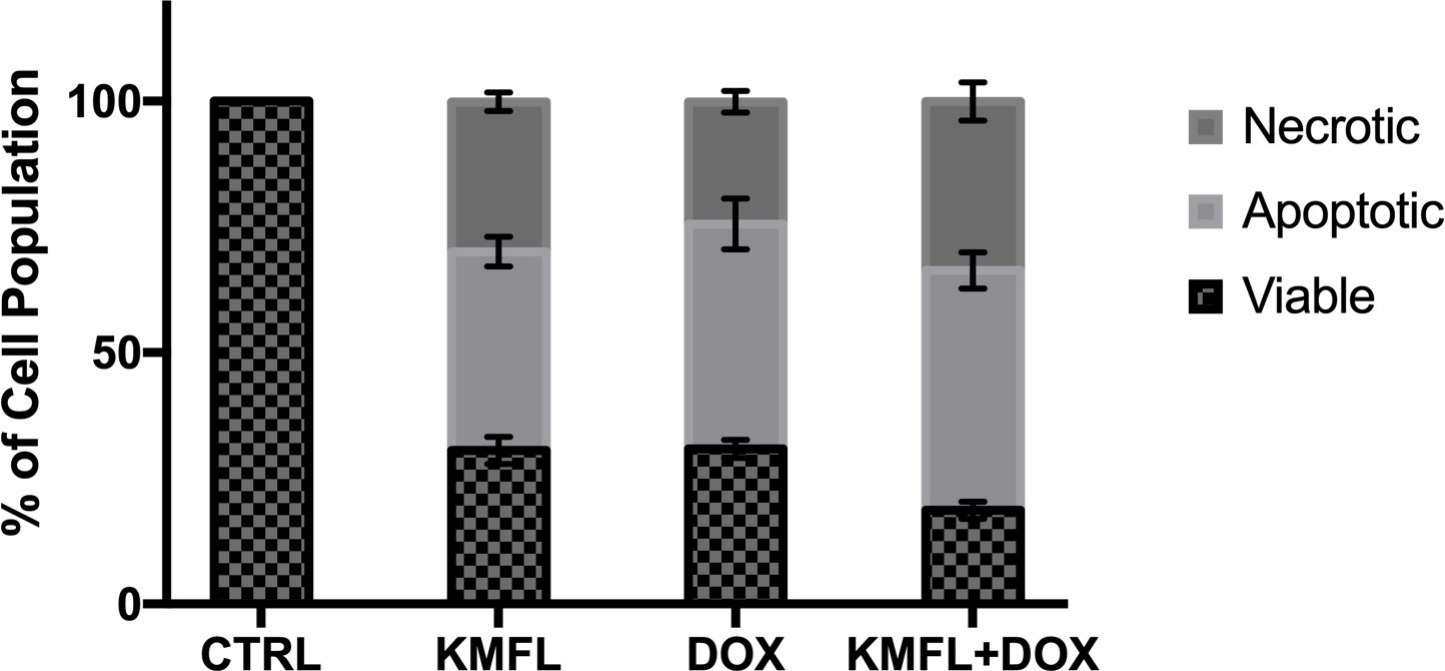
Mode of cell death of MCF-7 cells after incubation with kaempferol 3-O-β-D-rutinoside, doxorubicin, and doxorubicin/kaempferol 3-O-β-D-rutinoside combination expressed as a percentage of different cell population ± SD.

Doxorubicin/Kaempferol 3-*O*-*β*-D-rutinoside combination showed higher percentages of apoptotic and necrotic cells (47.7% ± 14.8 and 33.6% ± 9.8, respectively) compared to those of the individual samples of doxorubicin and kaempferol 3-*O*-*β*-D-rutinoside (Fig 5 and Table 3).

**Table 3:** The effect of kaempferol 3-*O*-*β*-D-rutinoside (10 μg/mL), doxorubicin (3 μg/mL), and doxorubicin/kaempferol 3-*O*-*β*-D-rutinoside combination on the mode of cell death of MCF-7 cells expressed as a percentage of different cell population ± SD.

Sample	Viable cells population (%)	Apoptotic cells population (%)	Necrotic cells population (%)
**Control (CTRL)**	100	--------	------
**Kaempferol 3-O-β-D-rutinoside (KMFL)**	30.5 ± 2.7	39.6 ± 2.9	29.8 ± 1.9
**Doxorubicin (DOX)**	30.8 ± 1.8	44.8 ± 5.1	24.3 ± 2.1
**Doxorubicin/kaempferol 3-O-β-D-rutinoside combination (KMFL+DOX)**	18.6 ± 1.7	47.7 ± 3.6	33.6 ± 3.8

Thus, kaempferol 3-*O*-*β*-D-rutinoside; the compound isolated from *Cassia glauca* leaves exerted a synergistic cytotoxic effect with doxorubicin on the MCF-7 cell line.

### Molecular docking study

Flavonols were previously reported as kinase enzymes inhibitors [60–63]. Diverse kinase enzymes are responsible for the activation of various transcription factors that are known to transcribe various inflammatory and carcinogenic markers, thus kinases inhibition may rationalize the cytotoxic activity [64, 65]. The overexpression and the increased activity of aldose reductase enzymes in different human cancers were also reported [66]. Aldose reductase enzymes were found to be involved in the tumorigenesis process via the production of growth factors that promote cell proliferation and inflammatory cytokines [17, 18, 67], thus aldose reductase activity inhibition could be evidence for cytotoxic effects. Aldose reductase inhibitors were found to be doxorubicin sensitivity enhancers as reported in the case of colon cancer cells and this can explain the synergistic effect on the cytotoxic activity with doxorubicin. They were also found as inhibitors for doxorubicin-induced inflammatory mediators produced in serum and heart guarding against the cardiotoxic effects of doxorubicin [22, 23, 68].

Thus, molecular docking of kaempferol 3-O-β-D-rutinoside; the compound isolated from *Cassia glauca* leaves; on RSK2 kinase (PDB: 3UBD) and AKR1B1 aldose reductase (PDB: 2IKI) enzymes were performed to rationalize its observed cytotoxic activity against HepG2 and MCF-7 cell lines as well as it synergistic cytotoxic effect with doxorubicin on MCF-7 cell line.

#### Validation of molecular docking algorithm

The molecular docking algorithm was initially validated by redocking of the co-crystallized ligands into the active site of the respective receptor with the calculation of root mean square deviation (RMSD) for reliability and reproducibility of the proposed docking algorithm [23]. SL0101 was docked to RSK2 (3UBD) while IDD388 was docked to AKR1B1 (2IKI) with RMSD less than 2.0 A° indicating a validated algorithm compared to the crystallographic structure [39, 41].

#### Interactions analysis with RSK2 (3UBD)

**Fig 6:**
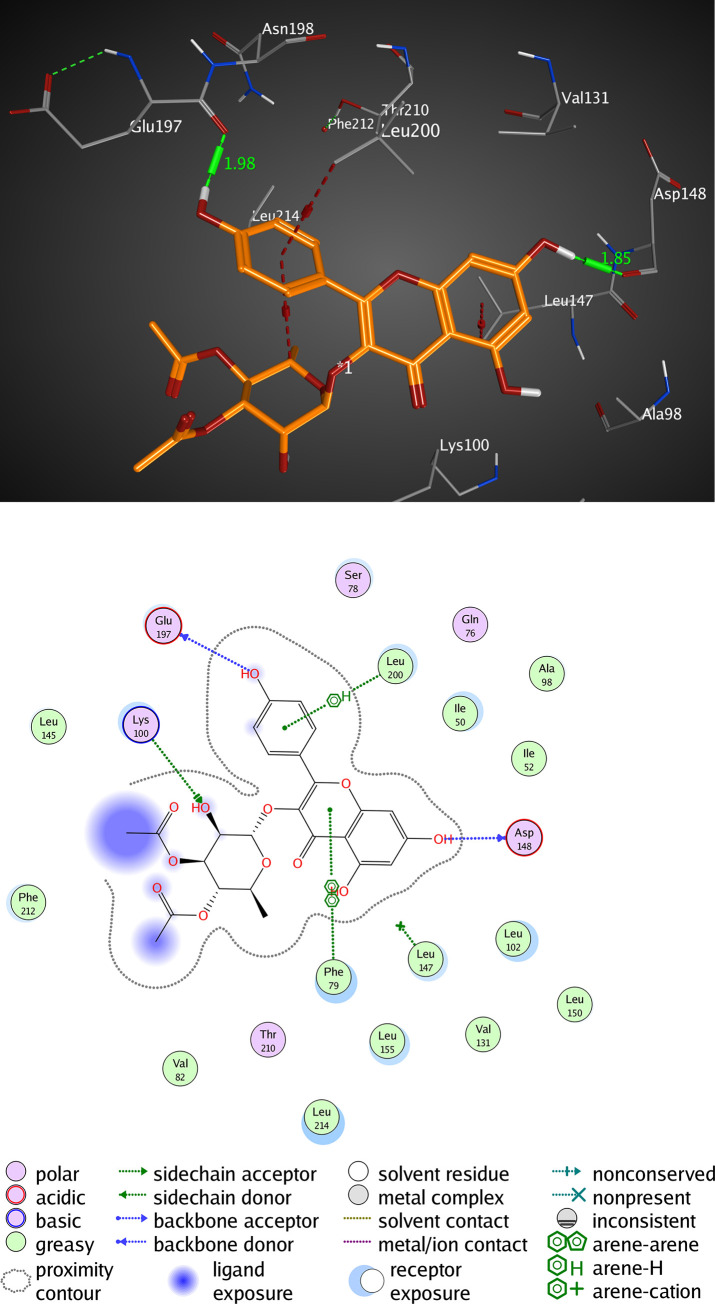
Molecular interactions of SL0101 co-crystallized with RSK2 (3UBD) as (A) 3D diagram (B) 2D diagram.

**Fig 7:**
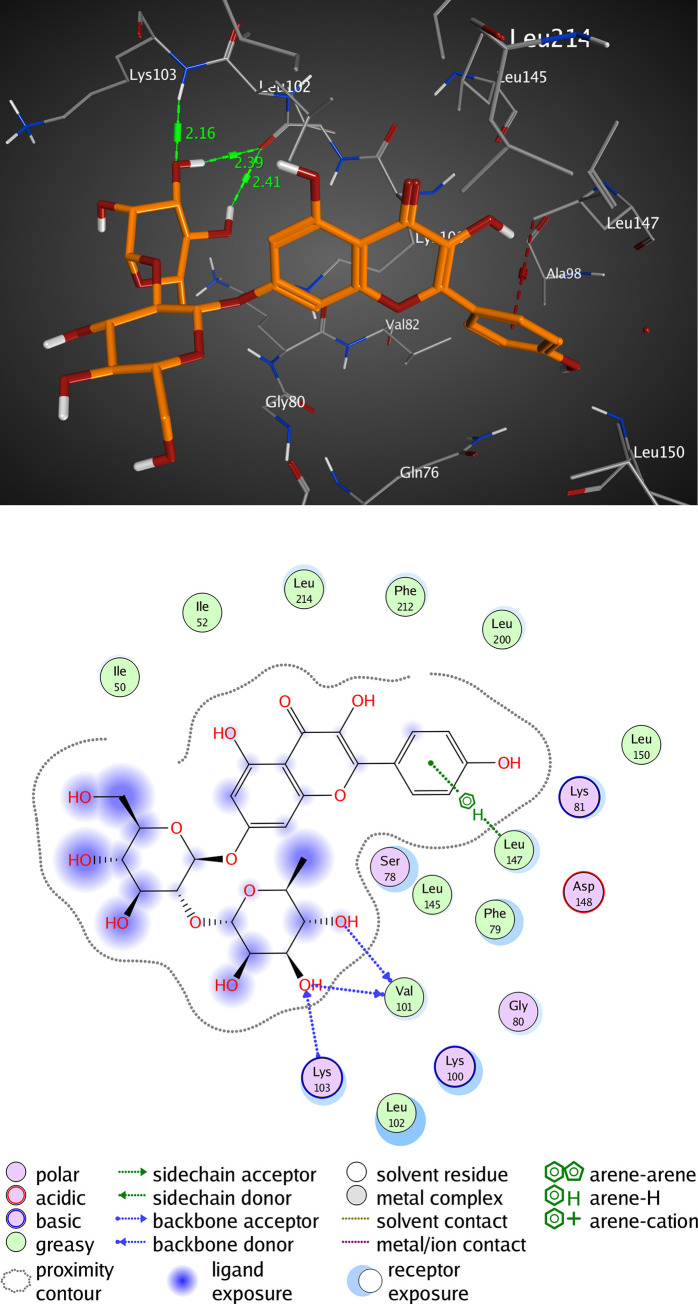
Molecular interactions of kaempferol 3-O-β-D-rutinoside with RSK2 (3UBD) as (A) 3D diagram (B) 2D diagram.

**Fig 8:**
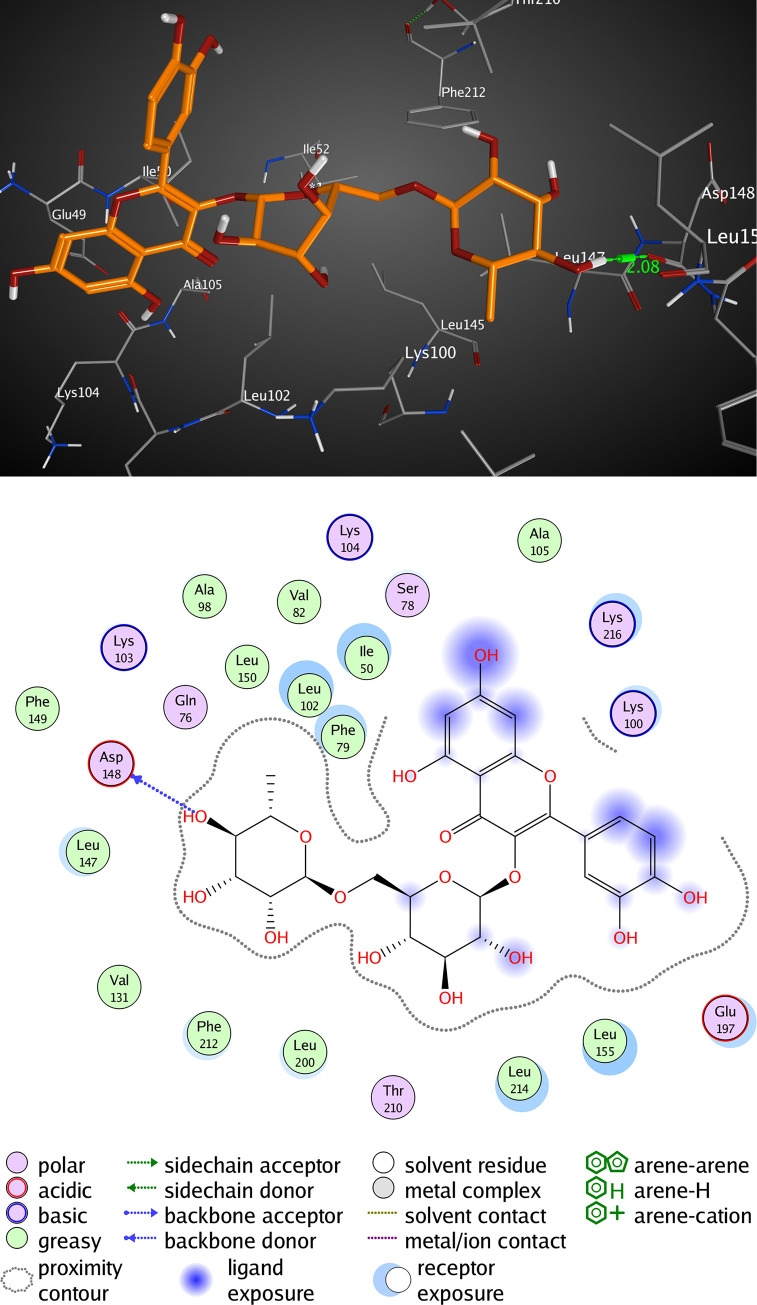
Molecular interactions of rutin with RSK2 (3UBD) as (A) 3D diagram (B) 2D diagram.

The active pocket of RSK2 (3UBD) consists mainly of PHE 79, LYS 100, VAL 101, LYS 103, LEU 147, ASP 148, GLU 197, and LEU 200, SL0101; the crystal ligand was interacted by three hydrogen bonds (H-H bonding) with LYS 100, ASP 148, and GLU 197, with distances of 1.94, 1.85, and 1.98 Å, respectively. The 5,7-dihydroxy-4H-chromene-4-one ring formed a hydrophobic interaction (arene-arene interaction) with PHE 79 with a distance of 3.16 Å while, the P-hydroxy phenyl moiety formed a van der Waal interaction (H-arene interaction) with LEU 200 with a distance of 3.04 Å. It showed an RMSD value of 1.669 Å, and a docking score of -8.311 kcal/mol (Fig 6 and Table 4). However, kaempferol 3-O-β-D-rutinoside interacted by two hydrogen bonds (H-H bonding) with VAL 101, and LYS 103 with distances of 2.16, and 2.39 Å, respectively. The P-hydroxy phenyl moiety formed a hydrophobic interaction (H-arene) with LEU 147 with a distance of 3.24 Å. It showed an RMSD value of 1.693 Å, and a docking score of -7.057 kcal/mol (Fig 7 and Table 4). Rutin interacted by one hydrogen bond (H-H bonding) with ASP 148 with a distance of 2.08 Å, RMSD value of 2.783 Å, and a docking score of -5.695 kcal/mol (Fig 8 and Table 4). Thus, kaempferol 3-O-β-D-rutinoside could potentially bind to the active site of RSK2 kinase enzyme with better docking score and RMSD value (-7.057 kcal/mol and 1.693 Å, respectively) compared to rutin (-5.695 kcal/mol and 2.783 Å, respectively) and relatively equal to the crystal ligand (-8.311 kcal/mol and 1.669 Å).

**Table 4:** Docking conformations of SL0101, kaempferol 3-O-β-D-rutinoside, and rutin with RSK2 (3UBD).

Ligand	RMSD value (Å)	Docking score (kcal/mol)	Interactions and Residues	Distance (Å)
**SL0101 (crystal ligand)**	1.669	-8.311	• H-H interactions:	
			LYS 100	1.94
			ASP 148	1.85
			GLU 197	1.98
			• π- π interactions:	
			PHE 79	3.16
			• π-H interactions:	
			LEU 200	3.04
**kaempferol 3-O-β-D-rutinoside**	1.693	-7.057	• H-H interactions:	
			VAL 101	2.16
			LYS 103	2.39
			• π-H interactions:	
			LEU 147	3.24
**Rutin**	2.783	-5.695	• H-H interactions:	
			ASP 148	2.08

#### Interactions analysis with AKR1B1 (2IKI)

**Fig 9:**
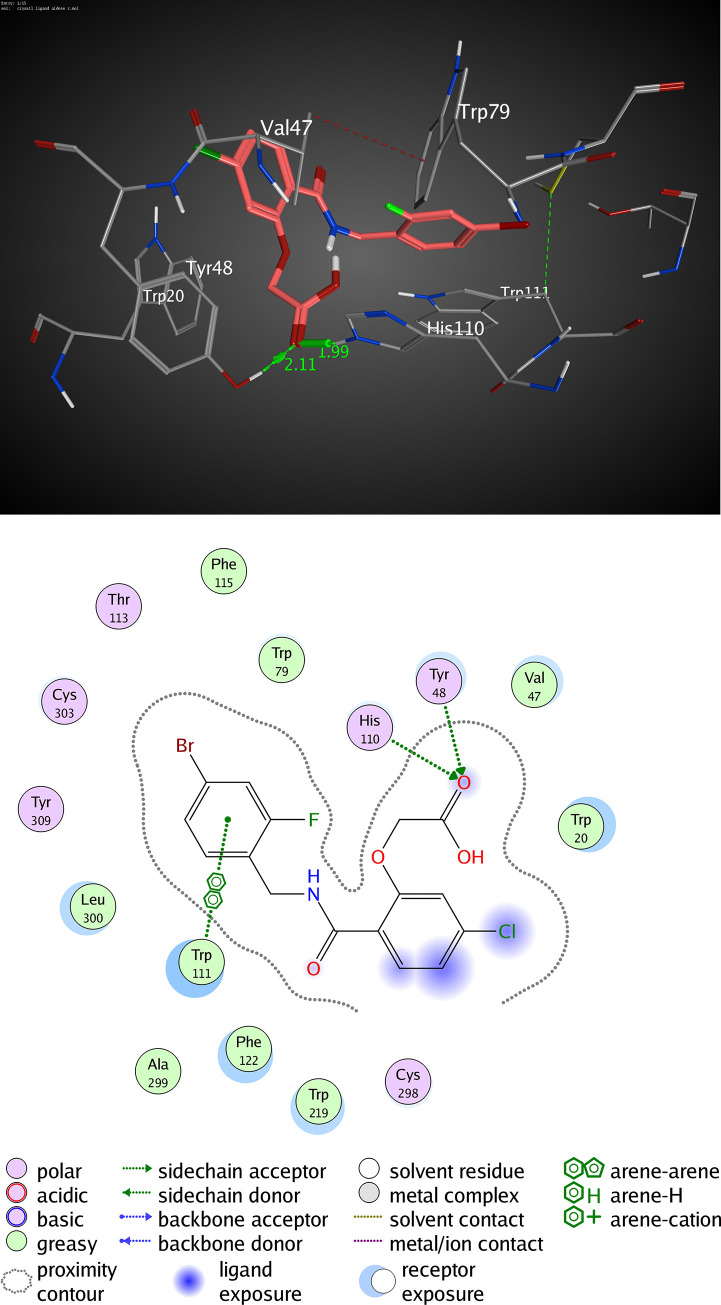
Molecular interactions of IDD388 co-crystallized with AKR1B1 (2IKI) as (A) 3D diagram (B) 2D diagram.

**Fig 10:**
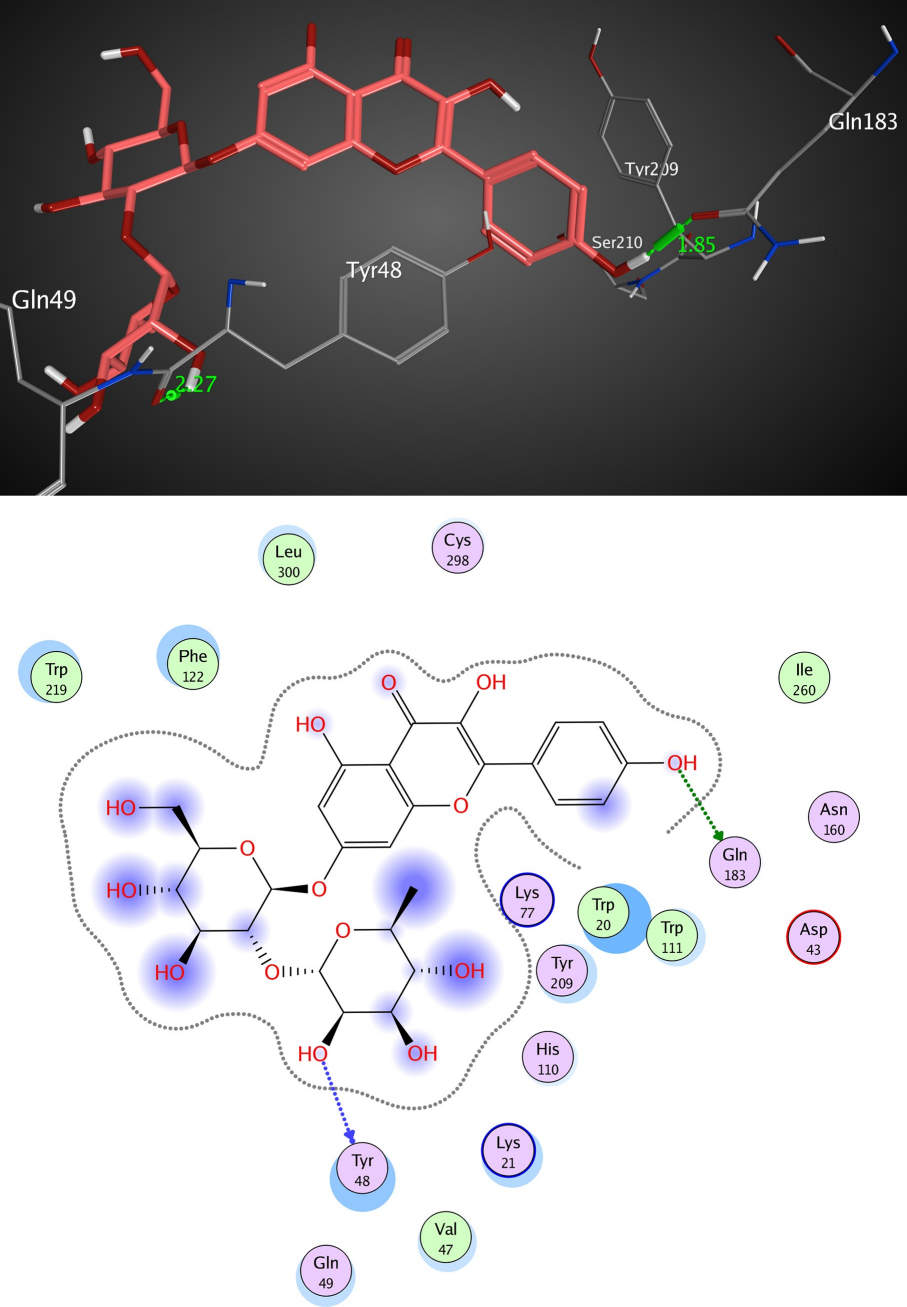
Molecular interactions of kaempferol 3-O-β-D-rutinoside with AKR1B1 (2IKI) as (A) 3D diagram (B) 2D diagram.

**Fig 11:**
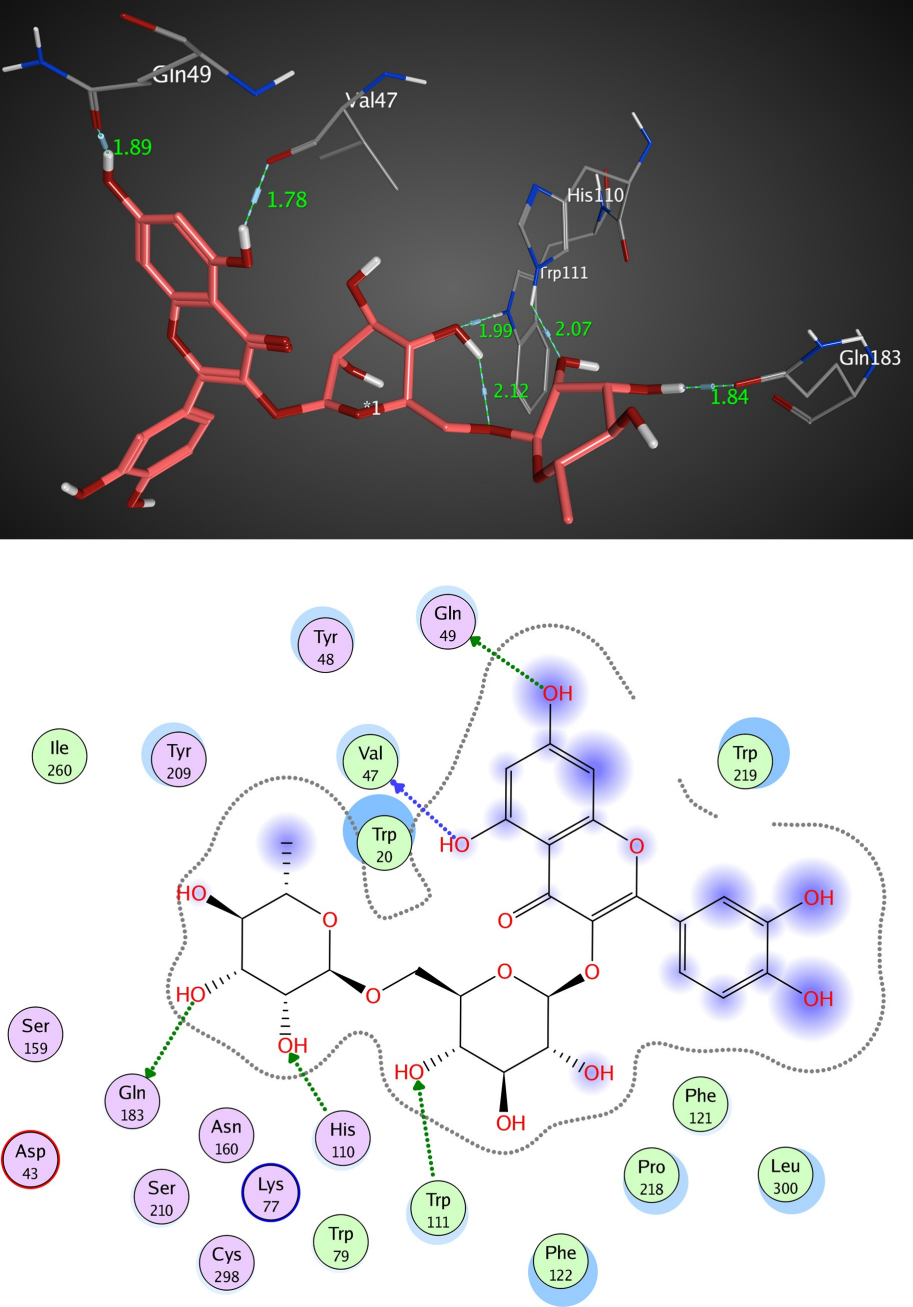
Molecular interactions of rutin with AKR1B1 (2IKI) as (A) 3D diagram (B) 2D diagram.

The active pocket of AKR1B1 (2IKI) consists mainly of VAL 47, TYR 48, GLN 49, HIS 110, GLN 183, and TRP 111. IDD388; the crystal ligand was interacted by two hydrogen bonds (H-H bonding) with TYR 48 and HIS 110 with distances of 2.11 and 1.99 Å, respectively. 1-Bromo-3-fluorobenzene ring interacted with TRP 111 by a π- π interaction with a distance of 2.47 Å. The RMSD value was 1.602 Å, and the docking score was -8.45 kcal/mol (Fig 9 and Table 5). Kaempferol 3-O-β-D-rutinoside was interacted by two hydrogen bonds of H-H interaction type with TYR 48 and GLN 183 with distances of 2.27 and 1.85 Å, respectively, with an RMSD value of 1.870 Å, and docking score of -7.47 kcal/mol. (Fig 10 and Table 5). Rutin interacted by five hydrogen bonds (H-H bonding) with VAL 47, GLN 49, HIS 110, TRP 111, and GLN 183 with distances of 1.78, 1.89, 1.99, 2.12, and 1.84 Å. The RMSD value was 2.901 Å with a docking score of -4.65 kcal/mol (Fig 11 and Table 5). Thus, also kaempferol 3-O-β-D-rutinoside could potentially bind to the active site of AKR1B1 aldose reductase enzyme having better docking score and RMSD value (-7.47 kcal/mol and 1.870 Å, respectively) compared to rutin (-4.65 kcal/mol and 2.901 Å, respectively) and relatively equal to IDD388 (-8.45 kcal/mol and 1.602 Å, respectively).

**Table 5:** Docking conformations of IDD388, kaempferol 3-O-β-D-rutinoside, and rutin with AKR1B1 (2IKI).

Ligand	RMSD value (Å)	Docking score (kcal/mol)	Interactions and Residues	Distance (Å)
**IDD388 (crystal ligand)**	1.602	-8.45	• H-H interactions:	
			TYR 48	2.11
			HIS 110	1.99
			• π- π interactions:	
			TRP 111	2.47
**kaempferol 3-O-β-D-rutinoside**	1.870	-7.47	• H-H interactions:	
			TYR 48	2.27
			GLN 183	1.85
**Rutin**	2.901	-4.65	H-H interactions:	
			VAL 47	1.78
			GLN 49	1.89
			HIS 110	1.99
			TRP 111	2.12
			GLN 183	1.84

#### Molecular mapping and flexible alignment determination

**Fig 12:**
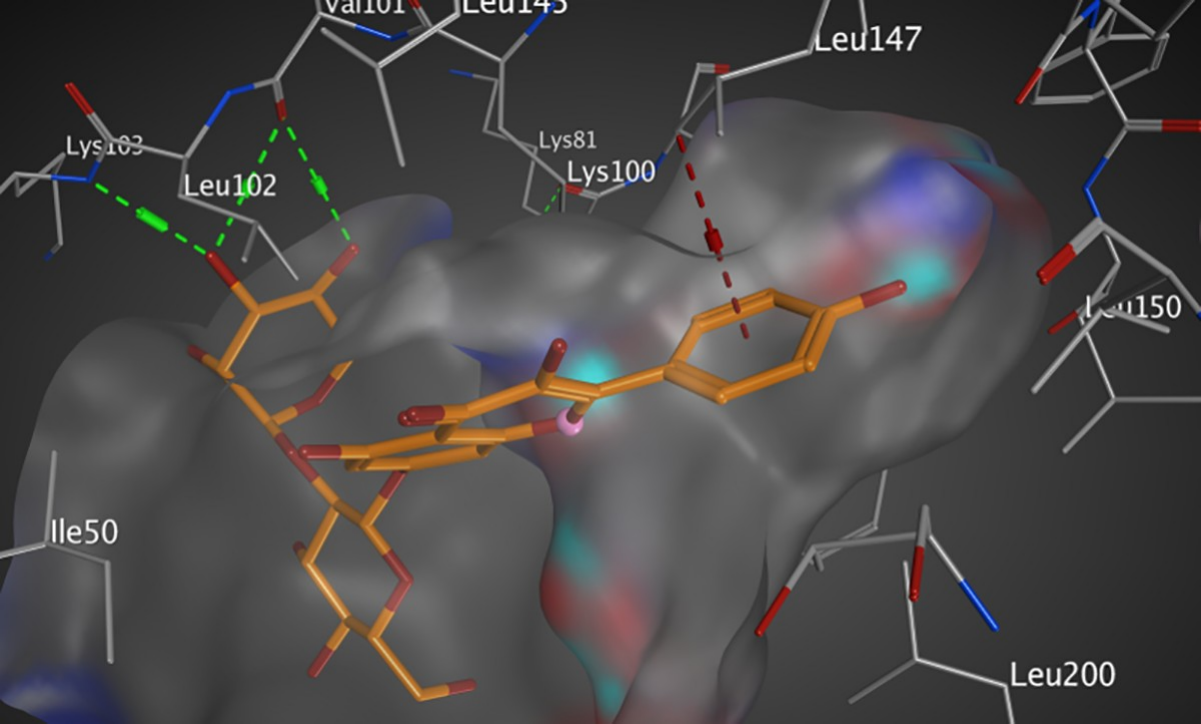
The molecular mapping of kaempferol 3-O-β-D-rutinoside interaction with RSK2 (3UBD) as a 3D diagram.

**Fig 13:**
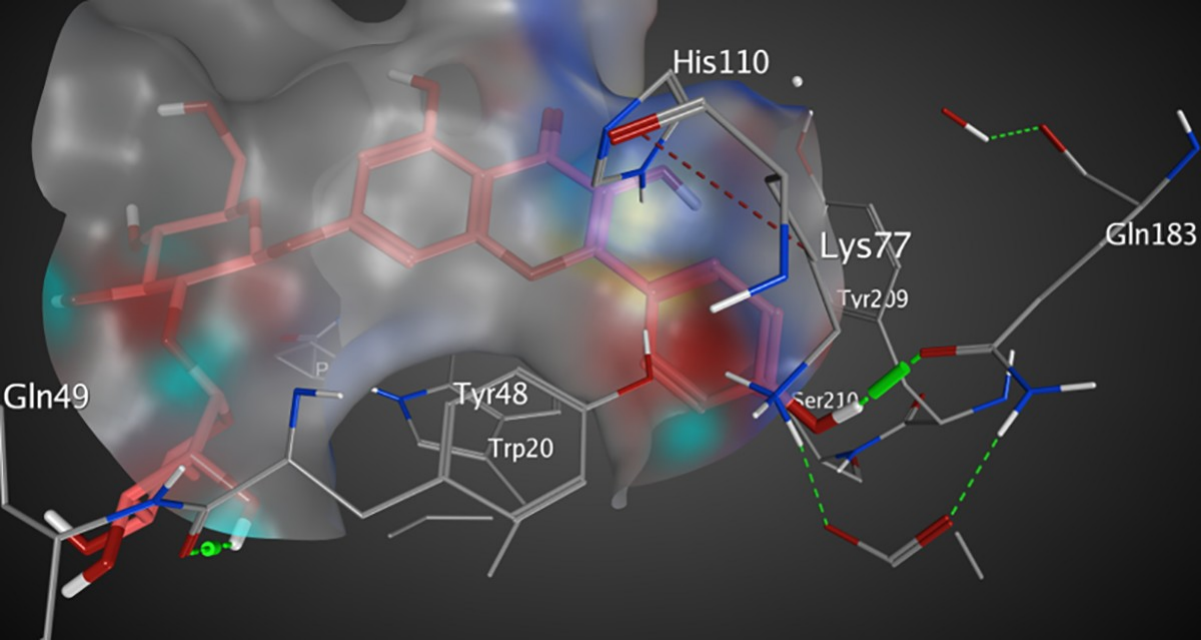
The molecular mapping of kaempferol 3-O-β-D-rutinoside interaction with AKR1B1 (2IKI) as a 3D diagram.

**Fig 14:**
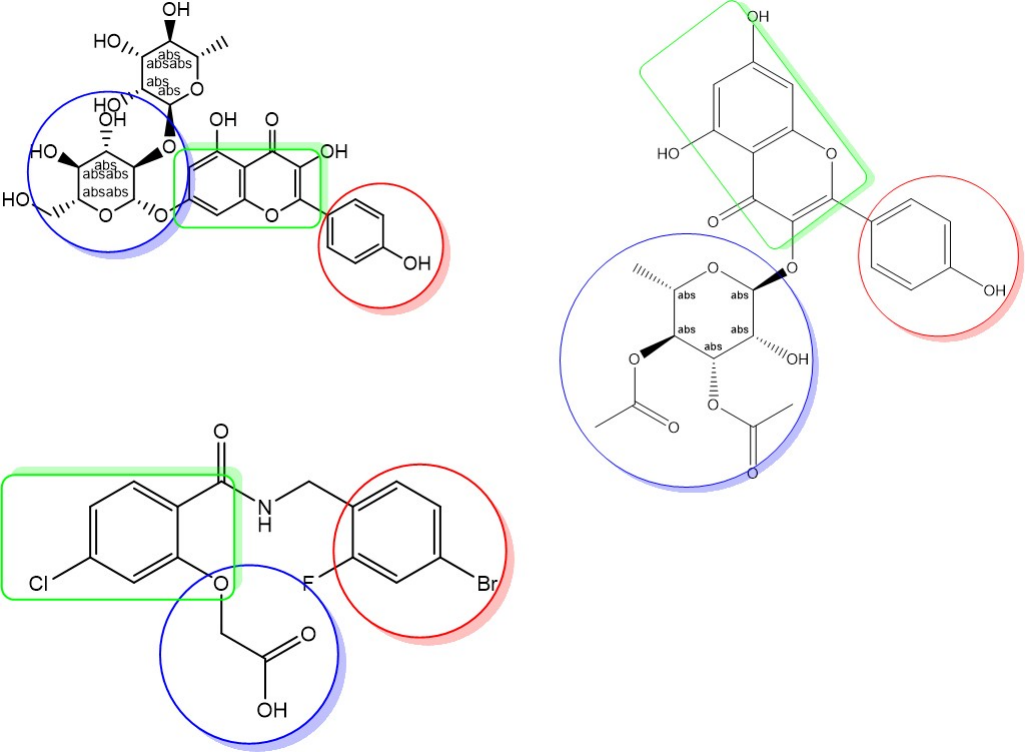
The common essential feature (the aromatic system; green-colored, the hydrophilic part; blue-colored, and the hydrophobic part; rose-red colored) between (A) Kaempferol 3-O-β-D-rutinoside (B) SL0101; the co-crystallized ligand of RSK2 (3UBD) (C) IDD388; the co-crystallized ligand of AKR1B1 (2IKI).

**Fig 15:**
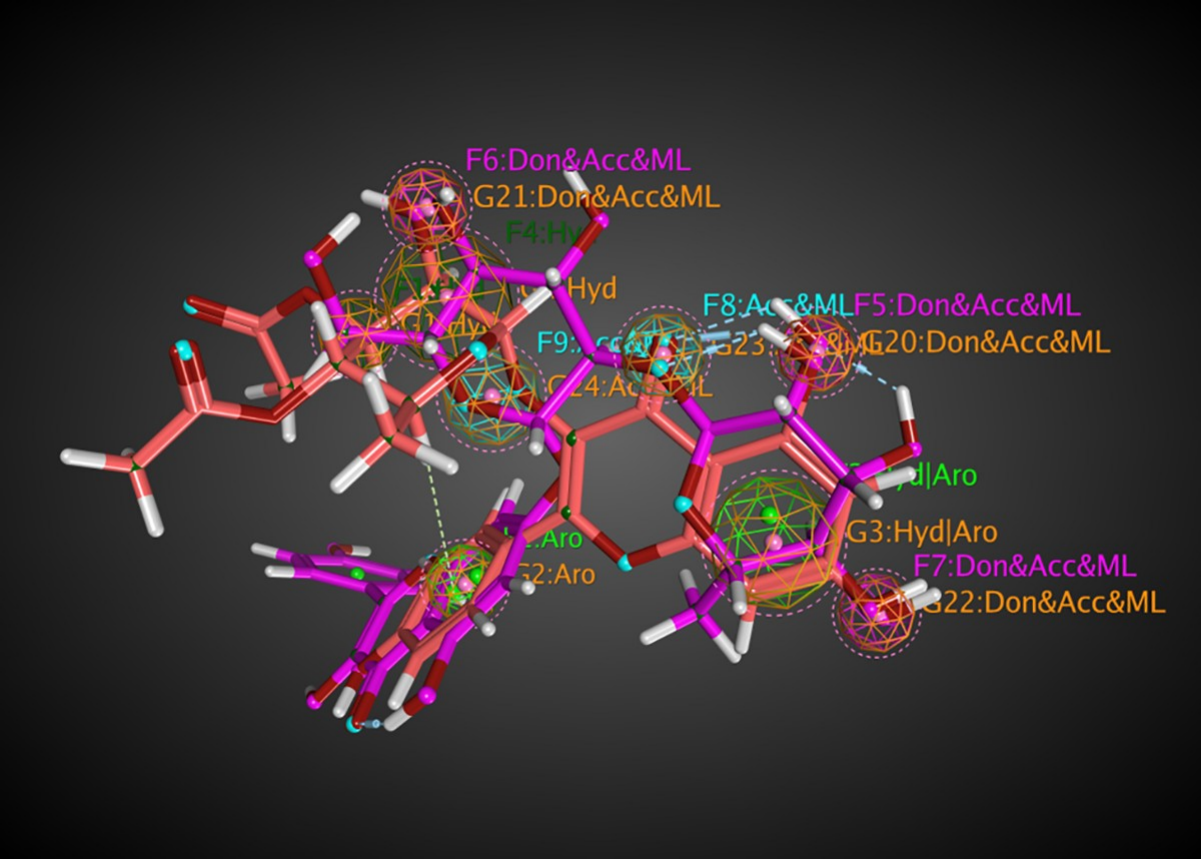
Flexible alignment between kaempferol 3-O-β-D-rutinoside (purple colored), and SL0101 (rose-red colored); the co-crystallized ligand of RSK2 (3UBD) as a 3D diagram.

**Fig 16:**
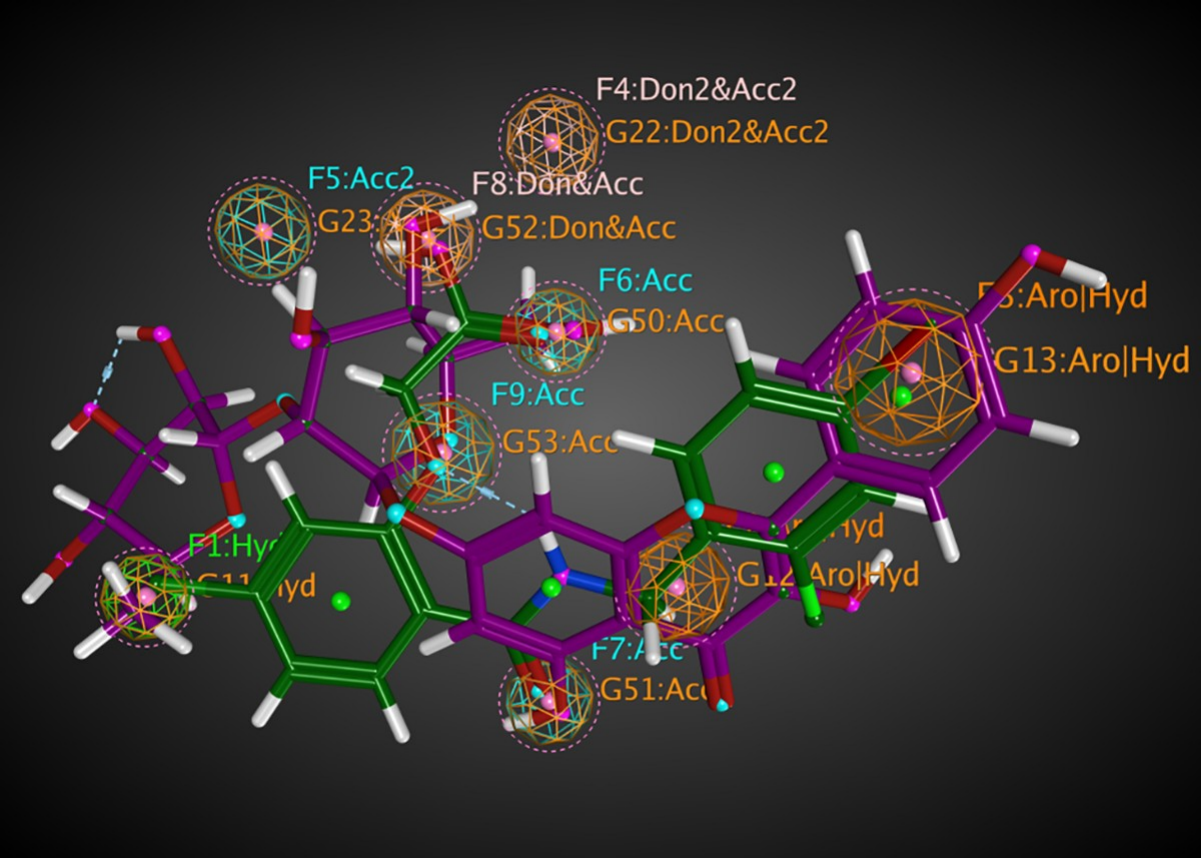
Flexible alignment between kaempferol 3-O-β-D-rutinoside (purple colored), and IDD388 (green colored); the co-crystallized ligand of AKR1B1 (2IKI) as a 3D diagram.

Molecular mapping of kaempferol 3-O-β-D-rutinoside with RSK2 showed a good occupying space in the targeted protein. The aromatic moiety occupying a hydrophobic region and forming PI-interaction with amino acid Leu147. The sugar part containing many hydroxy groups that act as a hydrogen bond acceptor-donator occupying the other part of the pocket and form three hydrogen bonds. While kaempferol 3-O-β-D-rutinoside with AKR1B1 showed excellent binding. Space was occupied by the aromatic planner moiety and attached to the targeted protein by two hydrogen bonds. Thus, molecular mapping confirmed that kaempferol 3-*O*-*β*-D-rutinoside is binding with the essential amino acids of RSK2 (3UBD) and AKR1B1 (2IKI) by occupying the original space of the crystal ligand and thus, the molecular docking processes were valid (Figs 12 and 13). Flexible alignment has been done between kaempferol 3-*O*-*β*-D-rutinoside, SL0101; the co-crystallized ligand of RSK2 (3UBD), and IDD388; the co-crystallized ligand of AKR1B1 (2IKI) to determine the essential feature of kaempferol 3-*O*-*β*-D-rutinoside compared to SL0101 and IDD388. It was found that kaempferol 3-*O*-*β*-D-rutinoside acquired common essential feature with both co-crystallized ligands represented by the presence of an aromatic system, a hydrophilic part, and a hydrophobic part (Fig 14). Moreover, it was found that kaempferol 3-*O*-*β*-D-rutinoside occupies the pockets of RSK2 (3UBD) and AKR1B1 (2IKI) better than the corresponding co-crystallized ligands (Fig 14). Furthermore, the pockets of RSK2 (3UBD) and AKR1B1 (2IKI) were found to have the same common essential feature. Thus, a good alignment between kaempferol 3-*O*-*β*-D-rutinoside and the co-crystallized ligands was confirmed (Figs 15 and 16).

## Conclusions

Kaempferol 3-*O*-*β*-D-rutinoside isolated from *Cassia glauca* leaves extract showed high cytotoxic activity against MCF-7 and HepG-2 cell lines. It also exerted a synergistic cytotoxic effect with doxorubicin on MCF-7 cell lines. Molecular docking study revealed that kaempferol 3-*O*-*β*-D-rutinoside exhibited binding scores to kinase and aldose reductase enzymes relatively equal to the standard selective inhibitors, and occupied their pockets better than the standard inhibitors, which rationalize its cytotoxic activity and expected protection against cardiotoxic effects of doxorubicin.

Thus, kaempferol 3-*O*-*β*-D-rutinoside could be used in combination with chemotherapeutic drugs to increase the sensitivity to their cytotoxic activity and protect against their side effects.

## Supporting information

S1 Fig MS/MS spectrum of quercetin glucoside at [M-H]^-^ 463 *m/z*. (TIF)

S2 Fig MS/MS spectrum of kaempferol rutinoside at [M-H]- 593 *m/z*. (TIF)

S3 Fig MS/MS spectrum of rutin at [M-H]^-^ 609 *m/z*. (TIF)

S4 Fig MS/MS spectrum of apigenin at [M-H]^-^ 269 *m/z*. (TIF)

S5 Fig Negative ESI-MS spectrum of Compound (1). (TIF)

S6 Fig Positive ESI-MS spectrum of Compound (1). (TIF)

S7 Fig ^1^H NMR spectrum of Compound (1). (TIF)

S8 Fig ^13^C NMR APT spectrum of Compound (1). (TIF)

S9 Fig Negative ESI-MS spectrum of Compound (2). (TIF)

S10 Fig Positive ESI-MS spectrum of Compound (2). (TIF)

S11 Fig ^1^H NMR spectra of Compound (2). (TIF)

S12 Fig ^13^C NMR APT spectrum of Compound (2). (TIF)

S1 File NMR spectral data for characterization of Compound (1). (PDF)

S2 File NMR spectral data for characterization of Compound (2). (PDF)
