# Supplementary figures and images for: Aldose reductase inhibitor form Cassia glauca: A comparative study of cytotoxic activity with Ag nanoparticles (NPs) and molecular docking evaluation
Source: PLoS One. 2020 Oct 16;15(10):e0240856. doi: 10.1371/journal.pone.0240856 (PMC7567391; doi:10.1371/journal.pone.0240856)

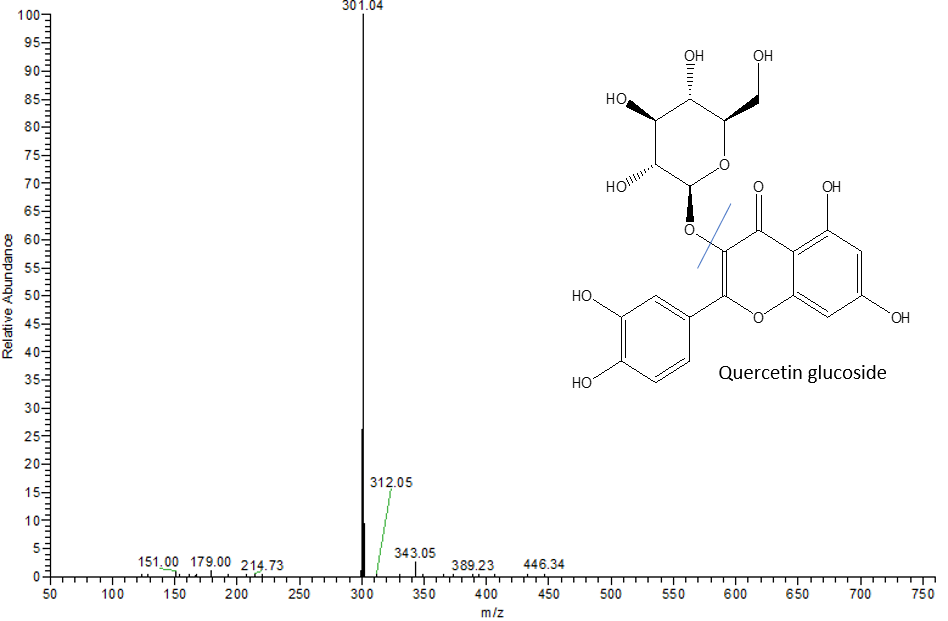

Supplement: S1 Fig — (TIF) [file pone.0240856.s001.tif]

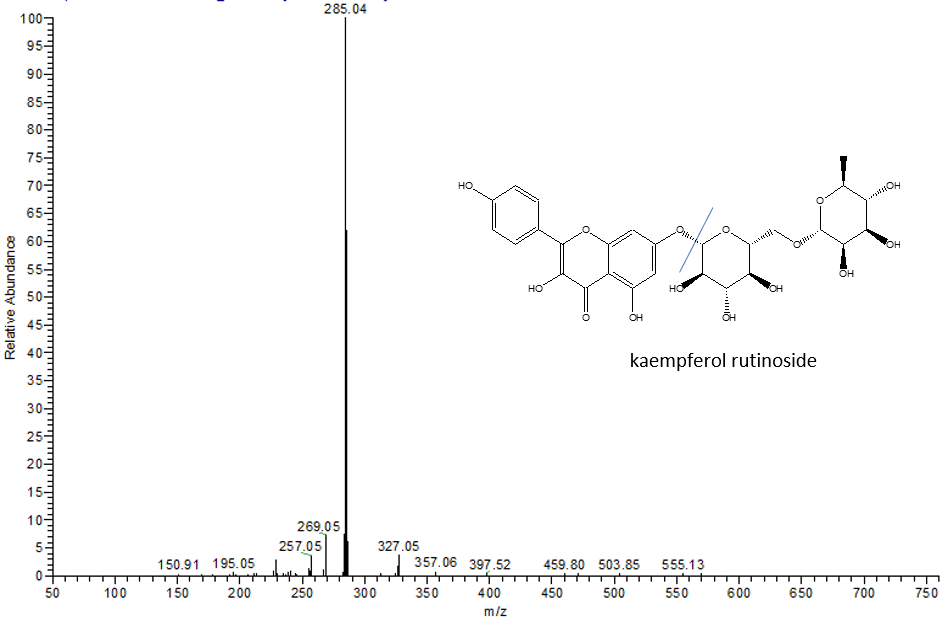

Supplement: S2 Fig — (TIF) [file pone.0240856.s002.tif]

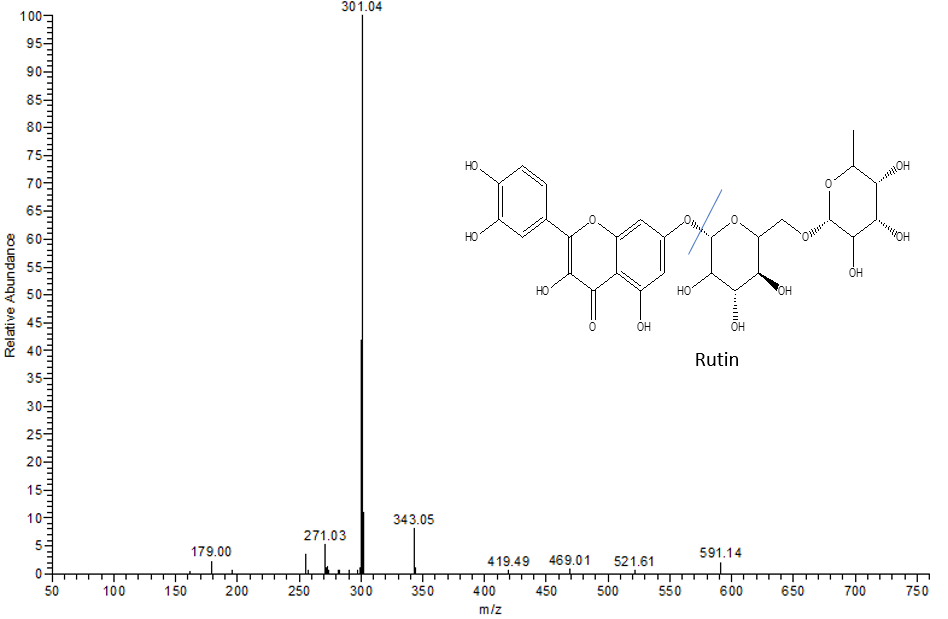

Supplement: S3 Fig — (TIF) [file pone.0240856.s003.tif]

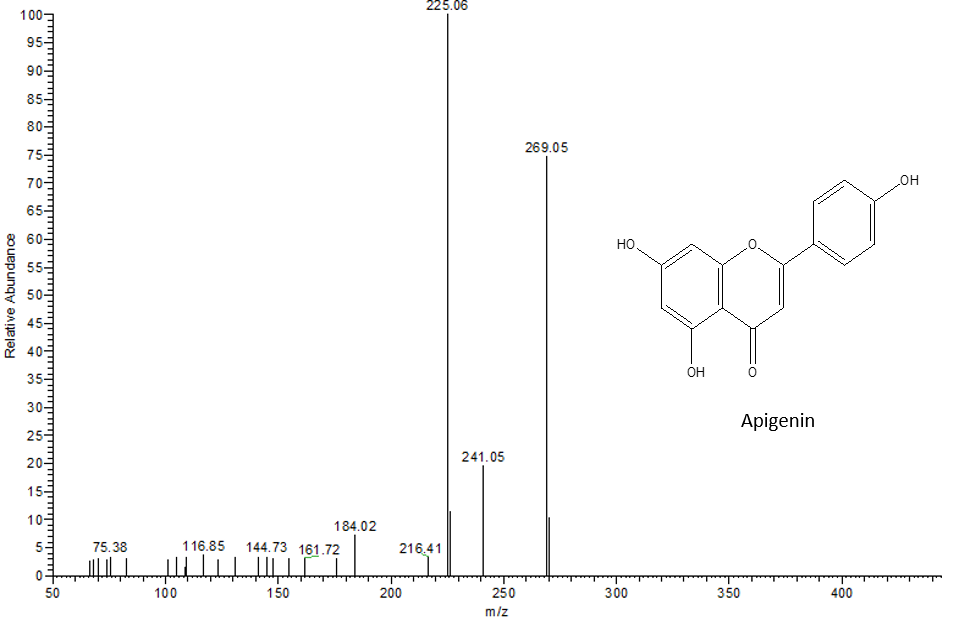

Supplement: S4 Fig — (TIF) [file pone.0240856.s004.tif]

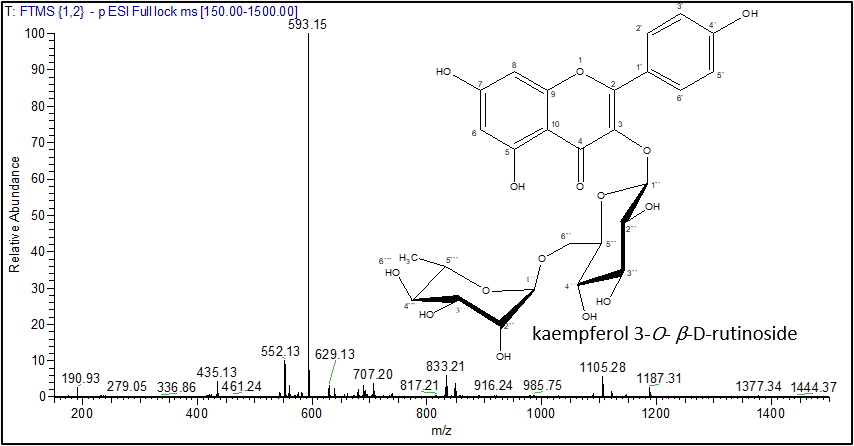

Supplement: S5 Fig — (TIF) [file pone.0240856.s005.tif]

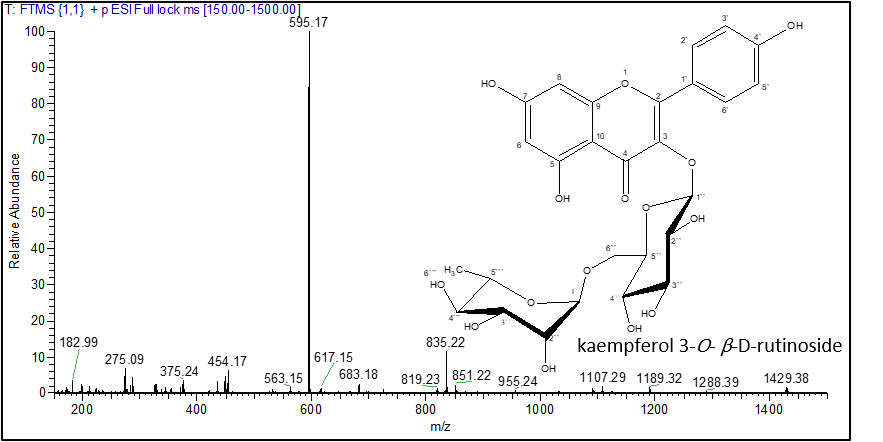

Supplement: S6 Fig — (TIF) [file pone.0240856.s006.tif]

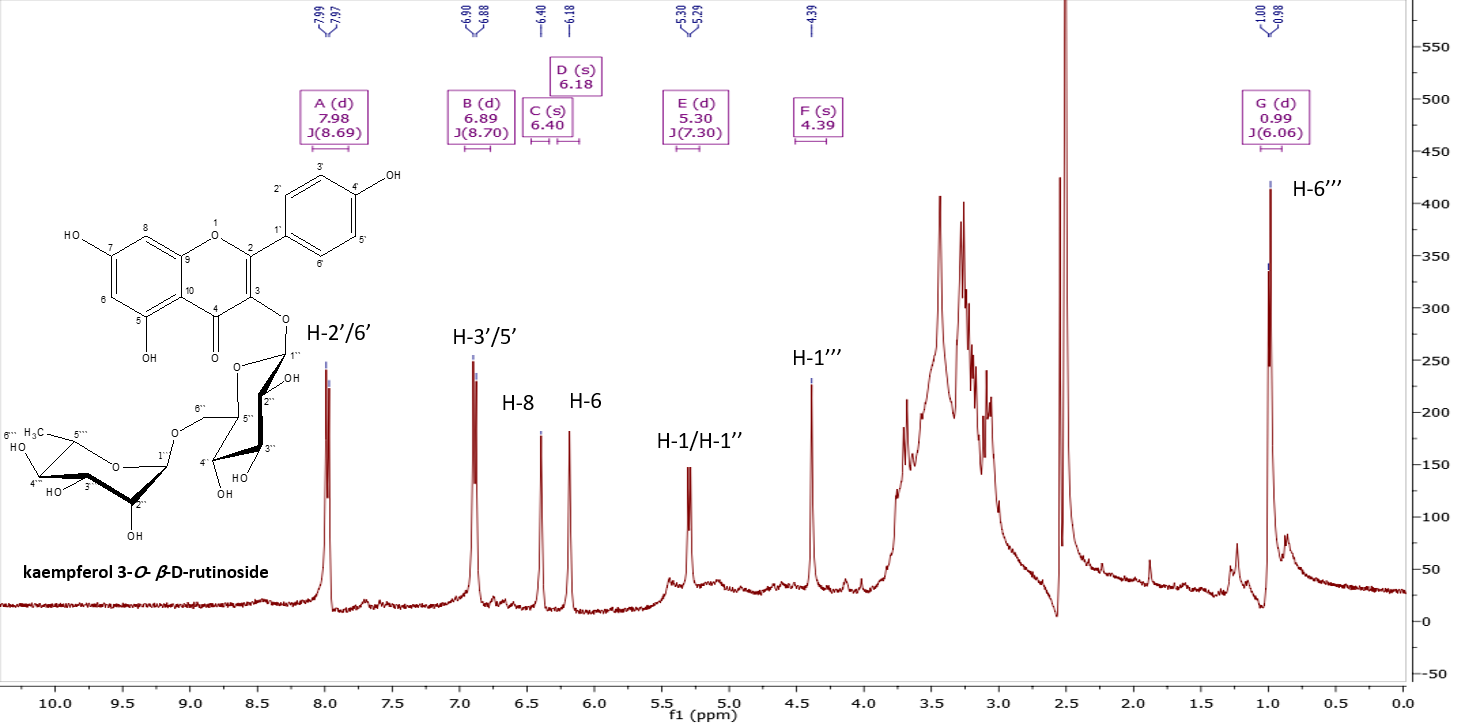

Supplement: S7 Fig — (TIF) [file pone.0240856.s007.tif]

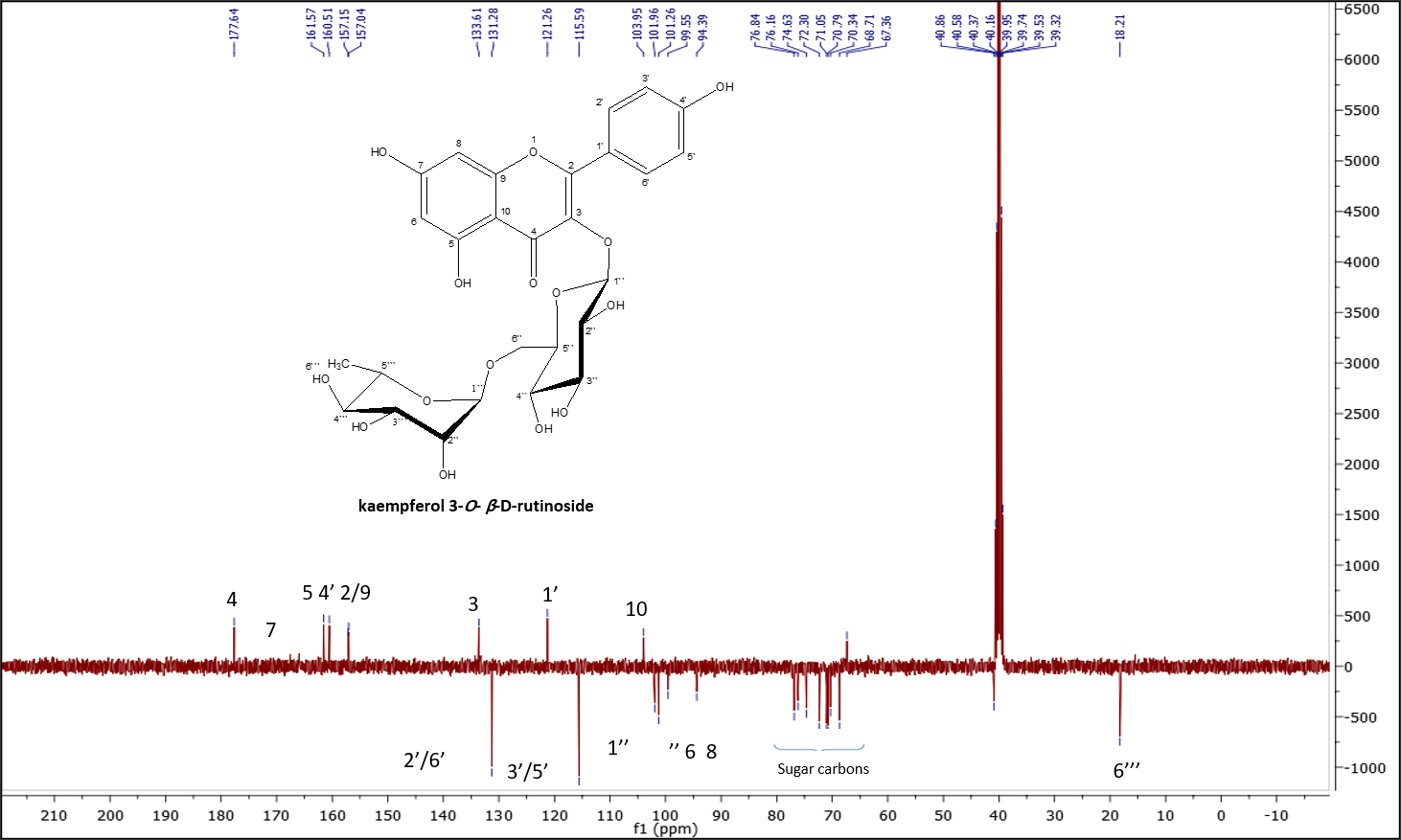

Supplement: S8 Fig — (TIF) [file pone.0240856.s008.tif]

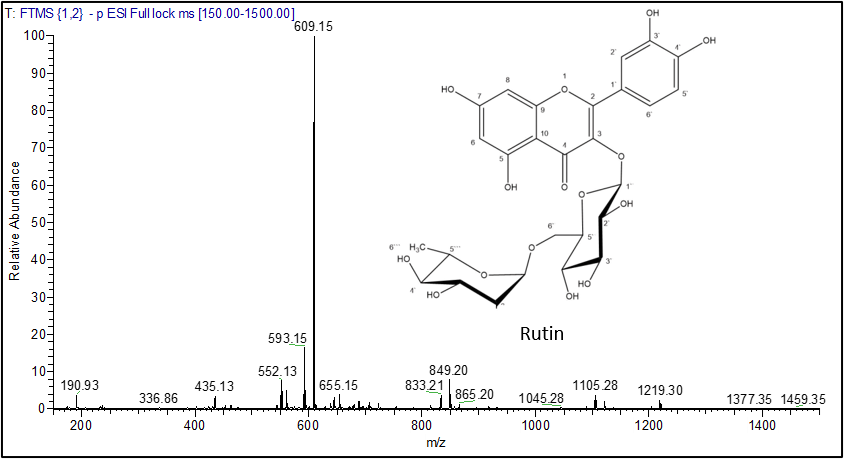

Supplement: S9 Fig — (TIF) [file pone.0240856.s009.tif]

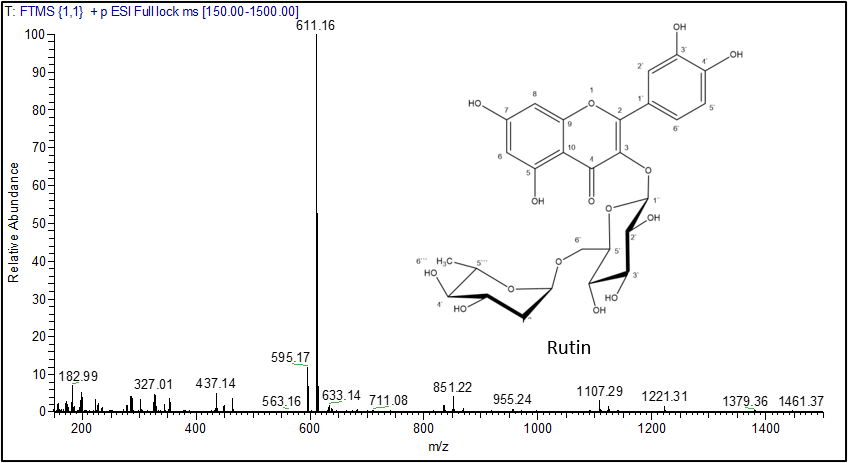

Supplement: S10 Fig — (TIF) [file pone.0240856.s010.tif]

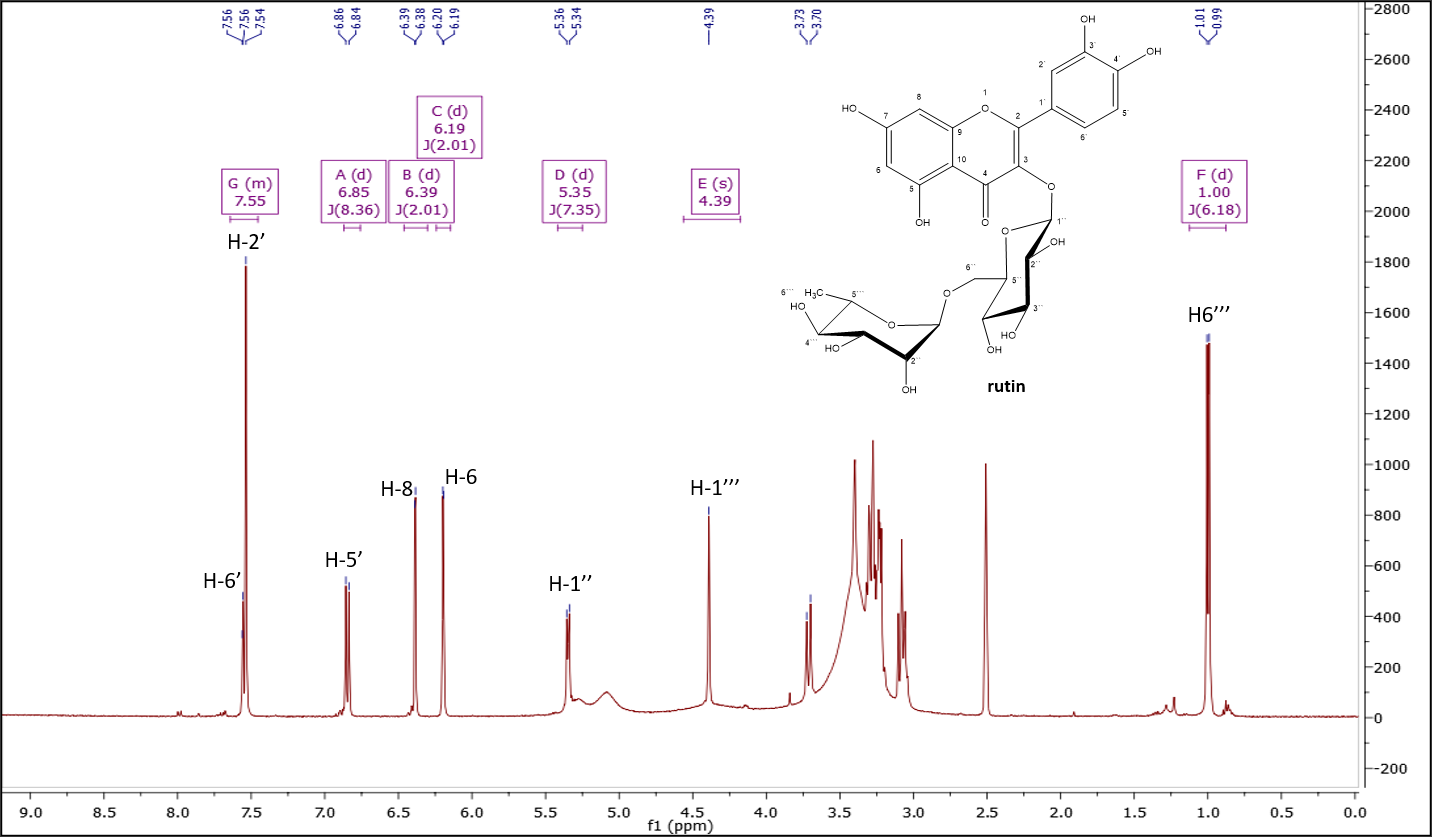

Supplement: S11 Fig — (TIF) [file pone.0240856.s011.tif]

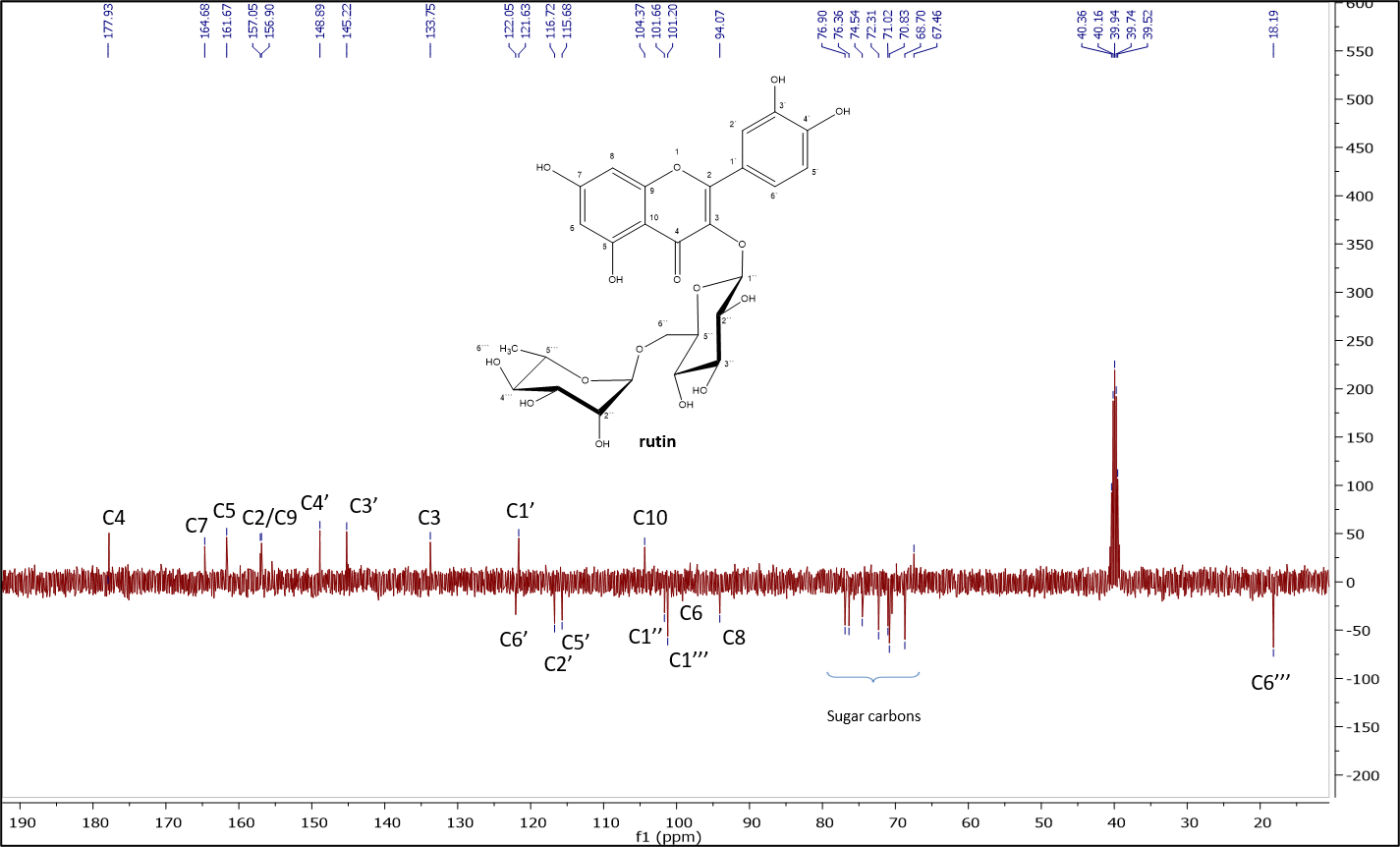

Supplement: S12 Fig — (TIF) [file pone.0240856.s012.tif]
