# Supplementary material for: Aldose reductase inhibitor form Cassia glauca: A comparative study of cytotoxic activity with Ag nanoparticles (NPs) and molecular docking evaluation
Source: PLoS One. 2020 Oct 16;15(10):e0240856. doi: 10.1371/journal.pone.0240856 (PMC7567391; doi:10.1371/journal.pone.0240856)
Supplement: S1 File — (PDF) [file pone.0240856.s013.pdf]

# NMR spectral data for characterization of Compound 1 (kaempferol 3-O-β-D-rutinoside)

## $^{1}$**H NMR** using JEOL JNM-ECA (400 MHz, DMSO-$d_6$)

δ ppm 7.98 (2 H, d, *J*= 8.69 Hz, H-2`/6`)

δ ppm 6.89 (2 H, d, *J*= 8.70 Hz, H-3`/5`)

δ ppm 6.40 (1 H, d, *J*= 2.02 Hz, H-8)

δ ppm 6.18 (1 H, d, *J*= 2.02 Hz, H- 6)

δ ppm 5.30 (1 H, d, *J*= 7.30 Hz, H-1``)

δ ppm 4.39 (1 H, brs, H-1```)

δ ppm 3.5-3.2 (m, remaining sugar protons)

δ ppm 0.99 (3 H, d, *J*= 6.06, H-6```)

$^{1}$H NMR spectrum of Compound (1) (**S7 Fig.**) exhibited an $A_2X_2$ spin coupling system of two *ortho* doublet, each integrated to two protons, at 7.98 (H-2`/6`) and 6.89 (H-3`/5`) indicated 4'-hydroxyl B-ring and AM spin coupling system of two meta doublets, each integrated for one proton at 6.40 (H-8) and 6.18 (H- 6) gave an evidence for 5,7-dihydroxy A-ring. In the aliphatic region the presence of rhamnosyl glucoside moiety was indicated from the presence of *β*-anomeric proton signal of glucoside moiety at δ ppm 5.30 (7.30 Hz) with a characteristic anomeric proton of terminal *α*-L-rhamnosyl at δ 4.39 together with signal of $CH_3$-6``` at δ 0.99 (6.06 Hz).

**$^{13}$C NMR APT** using JEOL JNM-ECA (400 MHz, DMSO-*d*$_6$)

δ ppm 177.64 (C-4)

δ ppm 166.16 (C-7)

δ ppm 161.57 (C-5)

δ ppm 160.51 (C-4`)

δ ppm 157.15 (C-2)

δ ppm 157.04 (C-9)

δ ppm 133.61 (C-3)

δ ppm 131.28 (C-2`/6`)

δ ppm 121.26 (C-1`)

δ ppm 115.59 (C-3`/5`)

δ ppm 103.95 (C-10)

δ ppm 101.96 (C-1``)

δ ppm 101.26 (C-1```)

δ ppm 99.55 (C-6)

δ ppm 94.39 (C-8)

δ ppm 76.84 (C-3``)

δ ppm 76.16 (C-5``)

δ ppm 74.63 (C-2``)

δ ppm 72.30 (C-4```)

δ ppm 71.05 (C-3```)

δ ppm 70.79 (C-4``)

δ ppm 70.34 (C-2```)

δ ppm 68.71 (C-5```)

δ ppm 67.36 (C-6``)

δ ppm 18.33 (C-6```)

$^{13}$C NMR APT spectrum of Compound (1) (**S8 Fig.**) exhibited thirteen $^{13}$C resonances of the kaempferol 3-*O*-glycoside moiety with key carbon signals of kaempferol nucleus at 177.64 (C-4), 166.16 (C-4`), 131.28 (C-2`/6`), 115.59 (C-3`/5`) and 133.61 (C-3). *O*-glycosidation at C-3 was indicated by down-field shift of C-2 at 157.15 and up-field shifted C-3 at 133.61. In the aliphatic region 12 carbon resonances of rutinoside moiety were assigned as 1```-6`` interglycosidic linkage that was deduced from down-field shift of C-6`` at 67.36 and up-field shift of C-5`` at 76.16.

Configuration of the sugar moieties was proved to be *β* and *α* pyranoses for D-glucose and L-rhamnose, respectively, on the basis of their δ and J-values in $^{1}$H and $^{13}$C NMR spectra.

All other resonances were assigned on the basis of their comparison with previous reported data (**Ref.: 50 & 51 in the manuscript**).
