# Supplementary material for: Aldose reductase inhibitor form Cassia glauca: A comparative study of cytotoxic activity with Ag nanoparticles (NPs) and molecular docking evaluation
Source: PLoS One. 2020 Oct 16;15(10):e0240856. doi: 10.1371/journal.pone.0240856 (PMC7567391; doi:10.1371/journal.pone.0240856)
Supplement: S2 File — (PDF) [file pone.0240856.s014.pdf]

NMR spectral data for characterization of Compound 2 (rutin)

^{1}H NMR using JEOL JNM-ECA (400 MHz, DMSO-d_6)

δ ppm 7.56 (1 H, H-6\`)

δ ppm 7.54 (1 H, d, *J*= 2.09 Hz, H-2\`)

δ ppm 6.85 (1 H, d, *J*= 8.36 Hz, H-5\`)

δ ppm 6.39 (1 H, d, *J*= 2.01 Hz, H-8)

δ ppm 6.19 (1 H, d, *J*= 2.01 Hz, H- 6)

δ ppm 5.35 (1 H, d, *J*= 7.35 Hz, H-1\`\`)

δ ppm 4.39 (1 H, brs, H-1\`\`\`)

δ ppm 3.43-3.06 (m, remaining sugar protons)

δ ppm 1.00 (3 H, d, *J*= 6.18 Hz, H-6\`\`\`)

^{1}H NMR spectrum of Compound (2) (**S11 Fig.**) exhibited an ABX spin coupling system of three resonances each integrated for one proton, attributable to H-2\`, H-6\` and H-5\` of 3\`,4\`-dihydroxy B- ring and AM spin coupling system of two *meta* doublets, each integrated for one proton at 6.39 (H-8) and 6.19 (H- 6) of 5,7-dihydroxy A-ring. In the aliphatic region the presence of rhamnosyl glucoside moiety was indicated from the presence of β-anomeric proton signal of glucoside moiety at δ ppm 5.35 (7.2 Hz) with a characteristic anomeric proton of terminal α-L-rhamnosyl at δ 4.39 together with signal of CH_3-6\`\`\` at δ 1.00 (6.18 Hz).

^{13}**C NMR APT** using JEOL JNM-ECA (400 MHz, DMSO-d_6)

δ ppm 177.93 (C-4)

δ ppm 164.68 (C-7)

δ ppm 161.67 (C-5)

δ ppm 157.05 (C-2)

δ ppm 156.90 (C-9)

δ ppm 148.89 (C-4`)

δ ppm 145.22 (C-3`)

δ ppm 133.75 (C-3)

δ ppm 122.05 (C-6`)

δ ppm 121.63 (C-1`)

δ ppm 116.72 (C-2`)

δ ppm 115.68 (C-5`)

δ ppm 104.37 (C-10)

δ ppm 101.66 (C-1``)

δ ppm 101.20 (C-1```)

δ ppm 99.62 (C-6)

δ ppm 94.34 (C-8)

δ ppm 76.36 (C-3``)

δ ppm 74.54 (C-5``)

δ ppm 72.31 (C-2``)

δ ppm 71.02 (C-4```)

δ ppm 70.83 (C-3```)

δ ppm 70.87 (C-2```)

δ ppm 70.44 (C-4``)

δ ppm 68.81 (C-5```)

δ ppm 67.46 (C-6``)

δ ppm 18.19 (C-6```)

^{13}C NMR APT spectrum of Compound (2) (**S12 Fig.**) exhibited fifteen ^{13}C resonances of the quercetin 3-*O*-substituted moiety with key carbon signals of quercetin nucleus at 177.72 (C-4), 149.54 (C-4`), 145.64 (C-3`), 122.05 (C-6`), 121.40 (C-1`), 116.80 (C-2`) and 115.96 (C-5`) (Agrawal 1989), in addition to signals assigned for rutinoside moiety characterized by down-field shift of C-6`` at 67.46 and up-field shift of C-5`` at 76.37, confirming 1```-6`` glycosidic linkage. Attachment of the rutinoside moiety to the aglycone part was deduced from up and down-field shift of C-3 and C-2, respectively.

Configuration of the sugar moieties was proved to be β and α pyranoses for D-glucose and L-rhamnose, respectively, on the basis of their δ and J-values in ^{1}H and ^{13}C NMR spectra.

All other resonances were assigned on the basis of their comparison with previous reported data (**Ref.: 52 & 53 in the manuscript**).
